# Effects of Sensor-Based, Site-Specific Nitrogen Fertilizer Application on Crop Yield, Nitrogen Balance, and Nitrogen Efficiency

**DOI:** 10.3390/s25030795

**Published:** 2025-01-28

**Authors:** Ludwig Hagn, Martin Mittermayer, Andreas Kern, Stefan Kimmelmann, Franz-Xaver Maidl, Kurt-Jürgen Hülsbergen

**Affiliations:** 1 Chair of Organic Agriculture and Agronomy, Technische Universität München, Liesel-Beckmann-Straße 2, 85354 Freising, Germany; martin.mittermayer@tum.de (M.M.); maidl@tum.de (F.-X.M.); kurt.juergen.huelsbergen@tum.de (K.-J.H.); 2 Field Crops Unit, Plant Technology Center, Technische Universität München, Dürnast 5, 85354 Freising, Germany; andreas.kern@tum.de (A.K.); stefan.kimmelmann@tum.de (S.K.)

**Keywords:** variable rate application, site-specific fertilization, proximal sensing, multi-spectral reflectance, nitrogen application

## Abstract

This study investigates the effects of sensor-based, variable-rate mineral nitrogen (N) application (VRA) in winter wheat (*Triticum aestivum* L.) on the spatial variability of grain yield, protein content, N uptake, N balance, and N efficiency compared with uniform N application (UA). To analyze the effects of VRA and UA on yield and N balance parameters, on-farm strip trials were conducted on heterogeneous arable fields covering an area of 49 hectares. The trials were carried out over a four-year period, from 2020 to 2023, with crops under both application methods placed in strips side-by-side. The N fertilizer requirements for growth stages (GSs) 32 and 39 were determined using an online map-overlay VRA method. This method integrated the site-specific yield potential and current plant development derived from spectral reflectance measurements using a tractor-mounted sensor system. The results show that the application of N fertilizer can be reduced by up to 38 kg ha^−1^ yr^−1^. The N efficiency can be increased by 15% and a significant reduction in variability of N balances can be achieved. However, the effects on yield and N efficiency are highly dependent on the specific application conditions (weather conditions, disease occurrence, and crop development). Not every field trial showed advantages of VRA over UA fertilization. Overall, the VRA system demonstrated encouraging potential, functioning as intended. However, further adjustment and optimization are required to ensure that the VRA fertilization system works robustly and reliably under on-farm conditions.

## 1. Introduction

### 1.1. Spatial Variability of Cropland

Spatial variability of cropland is driven by soil properties (e.g., texture, organic carbon, and nutrient content [1,2]), water availability, and erosion [3,4], leading to differences in yields and nitrogen uptake. With uniform N application, the spatial variability of grain yield may lead to N oversupply in low-yield zones associated with low N-use efficiency (NUE) and negative ecological effects due to N losses [5,6]. In addition, the yield potential in high-yield zones may not be fully realized.

A possible solution to this problem is site-specific N fertilization, which can have several benefits, including positive yield effects, more uniform plant stands and protein contents [7,8,9], and reduced fertilizer inputs and costs [10,11]. An increase in NUE can be achieved by redistributing or even reducing the fertilizer application according to the requirements of the crop stand and the site conditions [5,12]. This reduces N balance [13,14] and climate-relevant N emissions [15,16,17]. However, despite these potentially positive effects, there are still reservations and skepticism regarding the efficiency and benefits of site-specific fertilizer application [18,19]. Therefore, further studies are needed to clarify whether the positive effects mentioned can actually be achieved under practical conditions.

### 1.2. Research Needs

There are three main methods of variable-rate application (VRA) of N, including (1) map-based application using historical soil and yield data [20,21,22,23], (2) real-time (on-the-go, online) application [24,25,26], and (3) the combination of both online and map-overlay methods [27,28,29,30].

For map-based application, using various parameters (crop yield, soil properties, spectral reflectance data from drones or satellites [5,12,31,32]), and cluster algorithms (k-means, hierarchical clustering, or data fusion [10,23,33]), arable fields can be divided into homogeneous yield and management zones [34,35]. In addition to digitally determined yield parameters [36,37] and soil quality maps [38,39,40], farmers’ experience [23,41] can be taken into account to determine the site-specific yield potential. N application rates are determined based on the yield potential within each yield zone [42]. However, variations in weather conditions, actual plant development, and N uptake by the crop stand are neglected in this method [43,44].

Recent approaches have integrated artificial intelligence methods with current weather and satellite data to simulate nitrogen dynamics, enabling more precise, site-specific fertilizer recommendations [45,46].

Real-time (online) application: This fertilization system requires data on the development of the plant stand (biomass, N uptake) at the time of the fertilizer application, which can be recorded using a spectral reflectance measuring system [47] (e.g., a tractor-mounted multispectral sensor). Furthermore, fertilizer algorithms are required to determine the fertilizer application quantity depending on plant development and N uptake [48,49]. A major disadvantage of these systems is that the site-specific yield potential is not taken into account, which can lead to over-fertilization in low-yield zones of arable fields [22,50,51].

A combination of both VRA procedures (online + map overlay) can be advantageous for achieving high precision in VRA N application according to plant requirements and site conditions [27,28,29]. In some field experiments, higher N-use efficiency and lower N balances were found with this method compared with other VRA methods [14,27,52]. However, the results were mainly obtained in randomized plot experiments on homogeneous trial fields and not on heterogeneous arable fields under practical conditions [53].

Several commercial online application systems have been on the market for more than a decade, yet N application is still mostly carried out uniformly [24,54]. Although several studies indicate that VRA can lead to a reduction of fertilizer inputs without decrease in yield and thus, to a reduction in the environmental impact of N oversupply, farmers are still not convinced of the advantages of VRA systems [55,56]. This is mainly due to doubts about the economic benefits of the high investment costs for small-scale farm structures [57], but also the lack of evidence of positive effects on yield and N efficiency. This illustrates the necessity to evaluate VRA systems under realistic farming conditions and to independently validate their performance.

### 1.3. Study Aims

In this study, the effects of sensor-based site-specific N fertilization in winter wheat (*Triticum aestivum* L.) on the spatial variability of grain yield, protein content, N uptake, N balance, and N efficiency were analyzed compared with uniform N fertilization based on the legally prescribed fertilizer algorithms of the German Fertilizer Ordinance (GFO) [58]. Site-specific on-farm strip trials were conducted at three locations in the years 2021 to 2023. Considering the site-specific yield potential, spectral reflectance measurements were carried out using a tractor-mounted sensor system and N fertilizer requirement algorithms [29]. N fertilizer was applied to winter wheat at two growth stages (GS 32, GS 39, [59]) using the online + map-overlay VRA method. To analyze and compare the effects of VRA and uniform application (UA), the two application methods were placed side by side on heterogeneous arable fields in the form of strip trials.

A key innovation of this study is the practical on-farm design, conducted on heterogeneous cropland and accounting for spatial variability within fields. This study also addresses a significant research gap by independently validating the performance of a combined VRA (online + map-overlay) method that integrates site-specific yield potential with real-time crop development. In contrast to previous studies conducted on homogeneous experimental plot trials, this study evaluates the efficiency of VRA with regard to different heterogeneous fields, providing realistic insights into its benefits and limitations under practical growing conditions.

## 2. Materials and Methods

### 2.1. Site and Weather Conditions

Over a period of three years (2021 to 2023), investigations were conducted on arable fields in experimental stations and commercial farms at three sites. Heterogeneous arable fields at the research stations Dürnast (48°24′3″ N 11°38′42″ E, 425 m a.s.l., 30 km north of Munich, Site A) and Roggenstein (48°10′47″ N 11°18′50″ E, 480 m a.s.l., 20 km west of Munich, Site B) of the Technical University of Munich (Bavaria, Germany) and arable fields from commercial farms in the region of Burghausen (48°7′51″ N 12°44′5″ E, 450 m a.s.l., 80 km east of Munich, Site C) were selected.

The investigated fields were cultivated using conventional farming methods. The study fields were selected due to their locations at different sites, the availability of management data, their sufficient size, and their heterogeneous soil properties (Table 1).

The strip trials were carried out on field A1 in 2020/2021, on field B1 and field C1 in 2021/2022, and on field C2 in 2022/2023. Site A is located at the tertiary hill country of southern Germany, which is characterized by tertiary sediments overlain by a thin loess blanket. The texture of the Haplic Luvisols of Site A varied greatly from loamy sand to loamy clay. The soil type was predominantly loam. SOC content ranged from 1.2 to 2.1%. The 30-year mean annual precipitation was 813 mm and the temperature was 8.8 °C (Table S1).

Site B is located in the Munich gravel plain, an alluvial plain of gravel, sand and boulders formed over several glacial periods. A distinctive characteristic of field B1 was the considerable variation of soil conditions, which is a characteristic attribute of the soil at the research station (Figure S1). The field was found to be composed of 32% organic soil (gley) and 68% sandy loam (Cambisol). SOC content ranged from 2.3 to 8.7% (Table 1). This presents a major challenge for N management. The 30-year mean annual precipitation was 888 mm and the temperature was 8.9 °C (Table S2).

The main soil types at Site C were Cambisols of medium quality (silty loam). SOC content ranged from 1.1 to 2.1% in field C1 and from 1.4 to 1.8% in field C2. The 30-year mean annual precipitation was 849 mm and the mean annual temperature was 8.9 °C (Table S3).

The year 2021 was very wet with heavy rainfall in the months of May and June. The temperatures were about average. In 2022, the spring was dry and the summer months were warmer with less precipitation (12 to 25%) than the 30-year average. The year 2023 was characterized by a cold and wet spring, followed by a dry and hot period during the summer months of June and July.

The selection of sites with different locations and varying heterogeneity enabled the evaluation of the fertilizer systems and their extended applicability.

### 2.2. Farm Management

The farmers carried out all plant cultivation measures (tillage, sowing, and plant protection) uniformly on the trial fields, apart from N fertilization at growth stages GS 32 and 39 and harvesting. The crop cultivation measures, dates, and application quantities of agrochemicals are shown in Tables S4–S7.

The crop management carried out in the trials corresponded to the conditions of agricultural practice and complied with the legal requirements for fertilizer application. Integrated pest control measures were implemented to maintain healthy plant stocks. Fertilization at vegetation start (vs) in spring was carried out with a uniform application rate of mineral fertilizers containing N and other nutrients as needed. The first N application rate was determined according to the guidelines and algorithms of the German Fertilizer Ordinance, considering the soil mineral N stocks (0–60 cm) in spring. Variable-rate applications were carried out at booting (GS 32) and jointing (GS 39).

### 2.3. N Fertilization Systems

#### 2.3.1. Reference Fertilization System GFO, UA Method

The regulations and algorithms of the GFO (2020), which are legally prescribed in Germany and represent the maximum permitted application of fertilizer, were used as the reference system (UA). This system calculates the N fertilizer requirement with balance methods depending on the type of crop grown, the target yield, and the target protein content. Furthermore, various additions and deductions are taken into account depending on the site conditions, the soil mineral N stock at start of vegetation (SMNvs), the previous crop, and other factors [53,58].

#### 2.3.2. Online + Map-Overlay VRA Method

The N fertilization system used in the experiments combined the map-based application approach and the real-time (online) application approach (online + map overlay). In the following, the methodology for the derivation of the yield potential and the integration of real-time data (current N uptake of the plant stands) into the VRA method is presented in detail.

**Derivation of the yield potential map**

Each field had to be divided into homogeneous yield and management zones, with a realistic target yield being set for each zone. Firstly, the arable land was roughly categorized using existing soil maps and the farmers’ assessment of the yield potential of the management zones. These were used to check whether a sufficiently heterogeneous area was available for the application of the VRA method.

**Figure 1:**
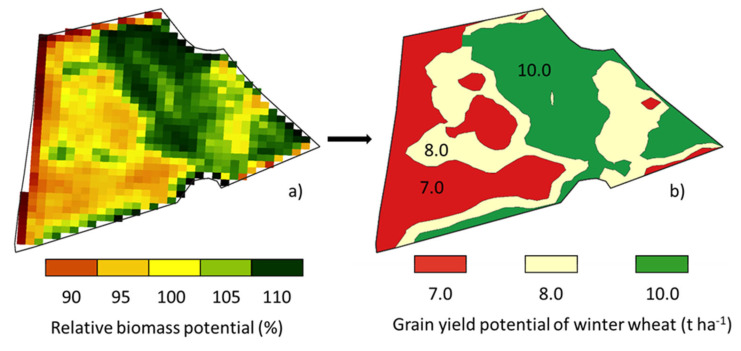
The rel. BMP map of field C1 (**a**) was converted to a yield potential map (**b**).

To determine the high-yield and low-yield zones of the fields more precisely, a satellite data-based approach was used—the “relative Biomass Potential” (rel. BMP)—according to a previously established methodology [31]. The rel. BMP was analyzed by processing a 5-year time series of Sentinel-2A images at crop-specific characteristic growth stages (for winter wheat: GS 32, GS 39, GS 65), using the “normalized difference vegetation index” (NDVI) as an indicator of aboveground biomass growth. The data were merged (Figure 1a) by calculating the mean values for all relevant GSs of crops across the 5-year time period. The rel. BMP of the study fields was divided into three homogenous zones (high, medium, and low yield) by quantile classification. The multi-year average yields, determined through measurements using a weighbridge, were defined as the target yields. Based on the average target yields, yield potentials in t ha^−1^ were assigned to the respective zones according to expert estimation.

This resulted in the yield potential map (example in Figure 1). The difference between the low-yield and the high-yield zones was 3.0 (t ha^−1^).

**Measurement of site-specific N uptake**

Plant development and N uptake on the fertilizer application dates was determined using a tractor-mounted multispectral sensor system (Compact Spec, [60]). The system was equipped with two multispectral sensors to record spectral reflection data in the wavelength range from 360 to 900 nm. The sensors were located 3 m from the center of the system (Figure S2). To compensate for variations in the intensity and spectral distribution of the sunlight, the natural solar radiation was recorded on a reference channel [16]. The spectral reflectance was recorded at one-second intervals. The data were collected and processed by a computer unit. A GPS device was used to record the georeferenced data points, with a deviation of less than 15 cm [52].

Custom-developed software (prefats, TUM, version 1.0.0.26) decoded the individual wavelengths at 10 nm intervals along with the corresponding spatial coordinates and calculated several predetermined vegetation indices, including the REIP. The REIP is the point of maximum gradient of plant reflectance [61,62] and is closely related to the aboveground biomass, N content, and N uptake of winter wheat [63,64,65]. Thus, the REIP index is key component for assessing nitrogen uptake and plant development in the VRA method.

(1) $$REIP=700+40(\frac{\frac{\left(670\;nm\;+\;780\;nm\right)}{2}-700\;nm}{740\;nm-700\;nm})$$

The determination of the current N uptake (Nup_cur_) at specific growth stages on the fertilizer application dates was carried out using measurement algorithms adapted to the REIP index by means of a linear equation. This equation was derived from fertilization trials with winter wheat conducted over a period of 20 years at different locations in southern Germany [29]:

(2) $${Nup}_{cur}=REIP\;*\;a+b$$

where *a* represents the gradient and *b* represents the axis intercept.

**Fertilizer algorithm (online + map-overlay VRA method)**

A fertilizer algorithm (Equation (3)), according to [29], was used in the online + map-overlay VRA method:

(3) $$FR=\left({Nup}_{nf}-{Nup}_{opt}+\left({Nup}_{opt}-{Nup}_{cur}\right)\right)\;*\;DIMA$$

The fertilizer requirement (FR) was calculated by comparing Nup_cur_ with Nup_opt_ (optimal N uptake at the current growth stage) at the respective growth stage. The optimum N uptake (Nup_opt_) was derived as a function depending on the development stage, yield potential, and protein content for winter wheat in field trials over several years with different levels of fertilizer application. The function describes the amount of N that the crop stand should have assimilated at a certain growth stage in order to achieve the target yield. Depending on the yield potential (e.g., 6, 8, 10 t ha^−1^), a specific N uptake function was used in the fertilizer algorithm. If Nup_cur_ < Nup_opt_, N fertilizer was applied according to the difference, if Nup_cur_ > Nup_opt_ no fertilizer was applied. The algorithm calculates the N requirement of the plant stand up to the next fertilizer application date (Nup_nf_) and adjusts the N application quantity accordingly.

The DIMA factor in Equation (3) accounts for environmental factors that influence the N use efficiency—(D) the duration of the effect of the fertilizer, (I) the immobilization rate of the N fertilizer in the soil, (M) the mineralization of soil N, and (A) the fertilizer utilization rate [14,29,66]. The DIMA factor was derived experimentally in fertilizer trials with wheat. The DIMA factor is crop- and location-specific. The algorithms (Equations (2) and (3)) were derived from and validated on datasets of several field trials.

The fertilization systems (VRA und UA) were implemented in a strip trial design to assess the comparison of their impact on yield, protein, nitrogen balance, and nitrogen efficiency.

**Adjustment of FR in the event of deviations from normal plant stands**

In the event of pronounced leaf changes or deviations from normal or healthy plant stands (e.g., due to plant diseases or leaf discoloration), the amount of N fertilizer was reduced accordingly in alignment with standard agricultural practices.

### 2.4. Experimental Design

To set up the strip trials, the tramlines first had to be recorded by a tractor-mounted GPS system. The fertilized plots were located between the tramlines of the study fields. The two fertilization systems (UA) and (VRA) were placed in every second tramline (Figure S3). The entire tramline was fertilized according to the respective fertilizer system. As entire fields were included in this study, the number of plots varied between the fields. The length of the plots was 40 m, while the width depended on the distance between tramlines. In fields A1, C1, and C2, the distance between tramlines was 15 m. At site B1, the distance was 24 m. Thus, the plots’ size varied between 600 and 960 m^2^. In addition, the length of the plots was aligned as much as possible with the yield potential map to avoid possible overlap with other yield zones. The plots were located in homogeneous zones of the fields.

The Nup_cur_ at GS 32 and GS 39 was measured for each plot before fertilization, using the tractor-mounted sensor system and the N-uptake algorithm adapted to the REIP [31]. Several georeferenced data points of the REIP were available for each plot, as a data point was recorded every second. For each plot, the REIP data points were averaged representing the N uptake for the entire plot (Figure S4). The data from the yield potential map were merged with the N uptake of the plots and the fertilizer algorithm was applied.

The fertilizer rates for the individual plots were illustrated on an application map. N was applied as calcium ammonium nitrate (CAN), using a hydraulic spreader with variable rate control by Kuhn (Rheinmünster, Germany) (Rauch Axis 30.2 H-EMC-W).

### 2.5. Crop Yield and Protein Data Collection

**Figure 2:**
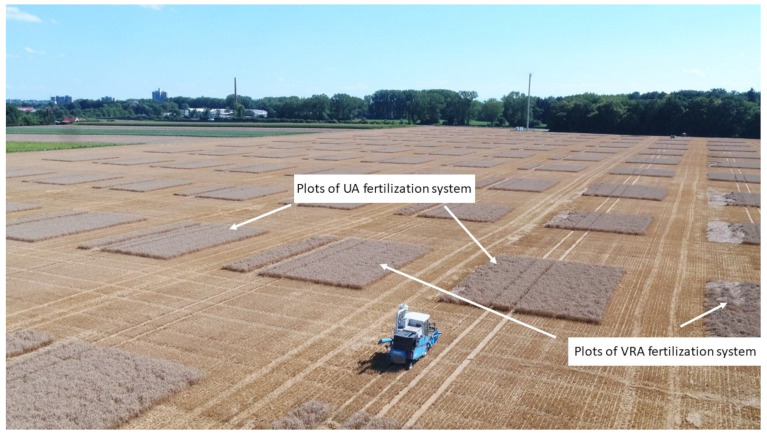
Grain yield harvest with a plot combine harvester and location of the plots on field B1, 2022.

The grain yield of each plot was measured by a Wintersteiger Delta plot combine harvester (Figure 2 and Figure S5). The grain yield was determined by three main components (real-time kinematic GPS positioning, determination of the harvested area, and determination of the grain moisture and the grain yield by means of weighing cells [67]). A grain yield sample was taken from each plot at harvest, weighed, and dried at 60 °C to determine the dry matter content (DM). Fresh matter (FM) yields (in t ha^−1^) were determined at 86% DM (Table S8). Samples were ground and N content and protein content were determined by a Vario Max cube C/N Analyzer. N uptake was calculated through multiplying grain yield by N content. Three distributed straw samples were taken from each yield zone and analyzed for N content in the laboratory. The straw yield was estimated based on a grain–straw ratio of 0.8 [68]. The straw was harvested at all sites and included in the N output.

### 2.6. N Balancing

The N surplus (kg ha^−1^) was determined using N balancing:

(4) $$N_{surplus}=N_{input}-N_{output}$$

where N_input_ refers to the amount of N applied from mineral and organic fertilizers (total N), N_output_ refers to the N content in the harvested biomass, including grain and straw, and N_surplus_ is an indicator of the potential loss of reactive N compounds such as ammonia (NH_3_), nitrous oxide (N_2_O), and nitrate (NO_3_^–^). The N efficiency (%) was calculated as follows:

(5) $$N\;efficiency=\frac{N_{output}}{N_{input}}$$

### 2.7. Statistical Analysis

The statistical data analysis was conducted using Rstudio software (version 2024.04.2). The statistical analyses were designed to quantify the effects of fertilization systems on the target parameters (e.g., yield, protein, N efficiency) and to identify significant differences between methods and yield zones. The following R packages were utilized: “stats, “agricolae”, “car”, “ggplot2”, and “FSA”. These packages were employed for the analysis of both parametric and non-parametric tests, given the lack of consistency of the data, where normality and homogeneity of variance were not always met [69].

The effects of the treatments (UA and VRA) on yield, protein content, N uptake, N balance, and N efficiency were tested across yield zones (high, medium, low). When the data demonstrated normal distribution and homogeneity of variances, standard ANOVA was performed followed by Tukey’s HSD post hoc test to identify specific group differences. In cases of violation of normal distribution and homogeneity of variance, the Kruskal–Wallis test was conducted, followed by Dunn’s post hoc test.

Boxplots were also part of the statistical analysis, illustrating the data distribution by quartiles, with the box representing the interquartile range (the middle 50% of the data) and the median indicated by a line inside the box. Whiskers extended to values within 1.5 times the interquartile range, while points beyond this range were considered outliers.

Datasets were separated for each yield zone. Significance of factors is indicated as follows: * *p* < 0.05, ** *p* < 0.01, *** *p* < 0.001.

## 3. Results

### 3.1. Field A1

**Figure 3:**
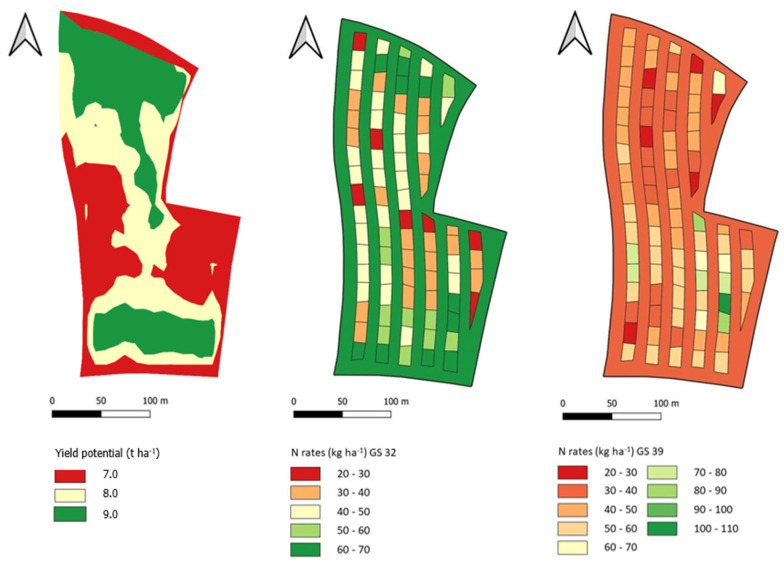
Yield potential map and N fertilizer application maps. Determined N fertilizer requirement and applied N fertilizer application rates at growth stages (GS) 32 and GS 39, field C1 (4.7 ha), winter wheat, 2021.

The distribution of N application rates under the VRA fertilization system at GS 32 was closely aligned with the yield zones (Figure 3). Higher N rates (50 to 70 kg ha^−1^) were applied in the high-yield zones and lower N rates (20 to 40 kg ha^−1^, shown in red) in the low-yield zones. At GS 39, the differentiation of the N rates between the yield zones was less pronounced. The amount of N applied with the UA fertilization system was relatively high at GS 32 (70 kg ha^−1^), while a substantially lower amount of N was applied at GS 39 (35 kg ha^−1^).

In field A1, the N fertilizer amount applied with the VRA fertilization system at GS 32 varied between 22 and 70 kg ha^−1^ and averaged 44 kg ha^−1^ (Table S9). The UA fertilization system according to GFO (2020) included application of 70 kg ha^−1^ mineral N. At GS 39, the fertilizer rates were between 18 and 114 kg ha^−1^. The mean N application rate was 49 kg ha^−1^. With the UA fertilization system, mineral N was applied at a rate of 35 kg ha^−1^.

**Figure 4:**
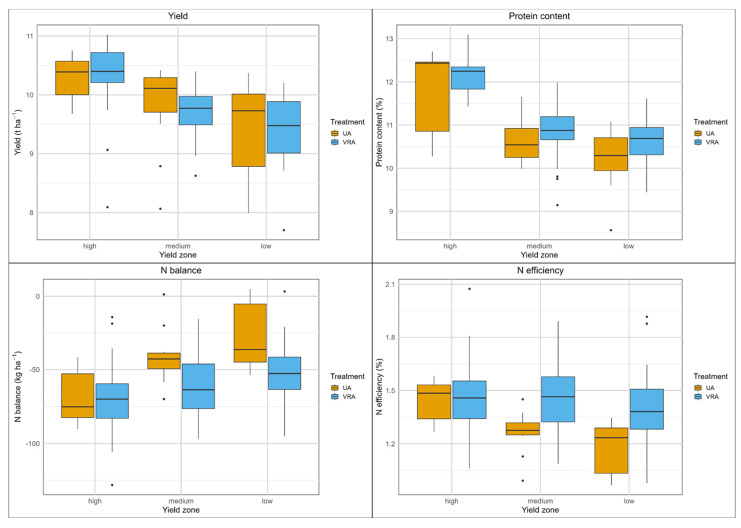
Boxplots of yield and N balance parameters, field A1, winter wheat, 2021.

The grain yield of the UA fertilization system varied between 8.0 and 10.8 t ha^−1^ (median of 10.1 t ha^−1^, Table S10), while the yield of the VRA fertilization system ranged from 7.7 to 11.0 t ha^−1^ (median of 9.8 t ha^−1^, Table S11). Significant differences (*p* < 0.05) in yield were found only in the medium-yield zone, where the UA fertilization system resulted in a 0.3 t ha^−1^ higher yield (Table 2). The measured yields exceeded the targeted yields in each yield zone by 1.4 to 2.5 ha^−1^. The highest yields from each fertilization system were recorded in the high-yield zones, exceeding those in than in the medium-yield zones, which in turn were higher than the yields in the low-yield zones, confirming the yield zone classification using the method employed (Figure 4).

There were considerable variations in N uptake (150 to 245 kg ha^−1^, UA fertilization system; 152 to 250 kg ha^−1^, VRA fertilizer system) and protein content (8.6 to 12.7%, UA fertilization system; 9.1 to 13.1%, VRA fertilizer system) (Tables S10 and S11). For both fertilization systems, the highest protein contents were found in the high-yield zone. N balances for both systems were predominantly negative, with a range of −90 to 5 kg ha^−1^ for the UA fertilization system and −128 to 3 kg ha^−1^ for the VRA fertilization system. Significantly lower N balances were demonstrated by the VRA fertilization system in the medium-yield zone (with a difference of 21 kg ha^−1^, *p* < 0.05) and in the low-yield zone (with a difference of 17 kg ha^−1^, *p* < 0.01). Both fertilization systems demonstrated remarkably high median N efficiencies, at 130 and 140%, respectively. The VRA fertilization system resulted in significantly higher N efficiencies in the medium-yield zone (18%, *p* < 0.01) and in the low-yield zone (15%, *p* < 0.01) compared with the UA fertilization system.

Overall, the UA fertilization system produced slightly higher yields, while the VRA system demonstrated higher protein content as well as higher N efficiency and lower N balance, due to the reduced amount of mineral fertilizer applied.

### 3.2. Field B1

The distribution of the N rates of the VRA fertilization system at GS 31 closely followed the pattern of the yield zones, with higher N rates (60 to 80 kg ha^−1^) applied in the high-yield zones and lower N rates (30 to 50 kg ha^−1^) in the low-yield zones (Figure S6). At GS 39, the spatial distribution of the N application rates did not correspond exactly to the yield zones but was more dependent on the current N uptake of the plant stand (and the REIP value of the sensor system).

The N application on field B1 at GS 31 resulted in a range of 31 to 73 kg ha^−1^ (mean 53 kg ha^−1^) when using the VRA fertilization system (Table S12). The application rate used in the UA fertilization system was 45 kg ha^−1^. At GS 39, the fertilizer rates ranged from 37 to 98 kg ha^−1^ (VRA fertilization system); the mean application rate was 65 kg ha^−1^. The UA fertilization system included application at a constant rate of 40 kg ha^−1^. This meant that significantly more mineral N was applied with the VRA fertilization system (on average 34 kg ha^−1^) than with the UA fertilization system. In this field, the fertilizer rates were not limited by GFO regulations when using the VRA fertilization system.

**Figure 5:**
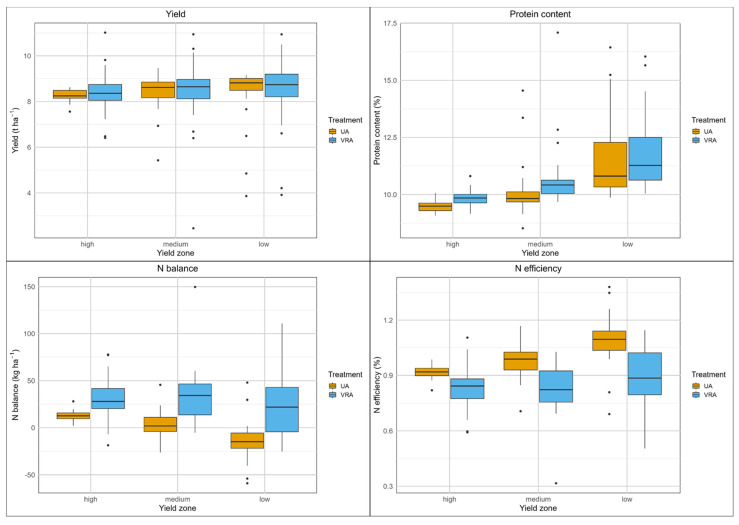
Boxplots of yield and N balance parameters, field B1, winter wheat, 2022.

The grain yield of the UA fertilization system varied considerably from 3.9 to 9.5 t ha^−1^, whereas the yield of the VRA fertilization system ranged from 2.5 to 11.0 t ha^−1^ (Tables S13 and S14). However, the boxplots, not including outliers, demonstrated- that the yield variation under both fertilization systems was not so substantial, ranging from 8.0 to 9.2 t ha^−1^ (Figure 5). No significant differences in yield were found between the fertilization systems (Table 3). The measured yields in the medium- and low-yield zones exceeded the targeted yields by 0.6 to 1.7 t ha^−1^, whereas in the high-yield zone, the measured yield of the VRA fertilization system was lower than expected (−0.6 t ha^−1^). Across all yield zones, the median yield was relatively consistent, ranging from 8.2 to 8.7 t ha^−1^. Notably, the highest yields were observed in the low-yield zone, indicating only minor differentiation among the yield zones.

Plant N uptake ranged from 107 to 214 kg ha^−1^ (UA fertilization system) and from 70 to 209 kg ha^−1^ (VRA fertilization system). Significantly higher plant N uptake (with differences of 8 and 6 kg ha^−1^) was found in the high- and medium-yield zones of the VRA fertilization system (*p* < 0.05 and *p* < 0.001, respectively), while no significant difference was observed in the low-yield zone. The mean N balance of the UA fertilization system was 0.6 kg ha^−1^. However, great variation was observed within the plots (−59 to 48 kg ha^−1^). The N balance under the VRA fertilization system varied extremely between −25 and 150 kg ha^−1^, with an overall mean of 29 kg ha^−1^. Significantly higher N balances (with differences of 15, 32 and 37 kg ha^−1^; *p* < 0.001, respectively) were observed in all yield zones of the VRA fertilization system. The protein content of both fertilization systems was rather low, with median values of 9.9% and 10.2%, but ranged from 8.5 to 17.1%. The VRA fertilization system resulted in significantly higher protein content in the high- and medium-yield zones (*p* < 0.001, respectively). It was noted that the protein content in the low-yield zone was considerably higher than in the high-yield and medium-yield zones, with a difference of up to 1.3 to 1.5%. In addition, the variability of protein content within the low-yield zone was much greater than in the medium- and high-yield zones for both fertilization systems.

The median N efficiency of the UA fertilization system was 100%, ranging from 70 to 140%. The median N efficiency of the VRA fertilization system was lower (80%), with a high variability of 30 to 110%. The N efficiencies of the UA fertilization system were significantly higher in all yield zones (*p* < 0.001, respectively).

Overall, the yields of both fertilization systems were similar, while the VRA system led to higher protein content and plant N uptake. However, significantly more fertilizer was applied by the VRA fertilization system, which negatively affected N balance and N efficiency.

### 3.3. Field C1

The N distribution within the VRA fertilization system was adjusted according to the yield zones, with higher N rates (40 to 60 kg ha^−1^) applied in the high-yield zones and lower N rates (20 to 30 kg ha^−1^) applied in the low-yield zones (Figure S7). At GS 43, the N application rates in the high-yield zones were minimal (0 to 10 kg ha^−1^), whereas in the low-yield zone, up to 20 kg ha^−1^ was applied. The VRA fertilization algorithm indicated that the plant N uptake in the high-yield zone was sufficient to reach the target yield; therefore, no further N fertilization was needed.

The amount of N applied under the VRA fertilization system showed considerable variation in field C1, ranging from 17 to 58 kg ha^−1^ at GS 32. The N rates at GS 43 had little variation (0 to 19 kg ha^−1^) (Table S15). The algorithm calculated fertilizer rates that were partially negative, indicating that the N plant uptake at GS 43 had already reached N_opt_ for achieving the targeted yield. The average N application rate under the VRA fertilization system was 37 kg ha^−1^, while that under the UA fertilization system was 50 kg ha^−1^ at GS 32. At GS 43, the mean N application rate used in the VRA fertilization system was 5 kg ha^−1^, while the uniform N rate was 30 kg ha^−1^.

On average, 168 kg ha^−1^ was applied according to the VRA fertilization method. The N fertilization of the UA fertilization system was 206 kg ha^−1^. Accordingly, N inputs could be reduced on average by 38 kg ha^−1^ and in partial zones by up to 63 kg ha^−1^. In this field, the fertilizer rates were not limited by GFO regulations when using the VRA fertilization system.

**Figure 6:**
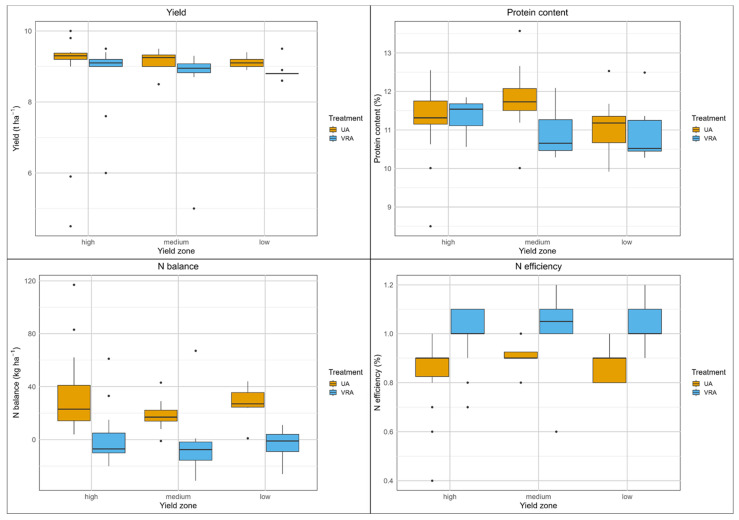
Boxplots of yield and N balance parameters, field C1, winter wheat, 2022.

The grain yield of the UA fertilization system varied between 4.5 and 10.0 t ha^−1^ (median of 9.2 t ha^−1^, Table S16), while the yield of the VRA fertilization system ranged from 5.0 to 9.5 t ha^−1^ (median of 9.0 t ha^−1^, Table S17). However, according to the boxplots, excluding the outliers, the yield was fairly consistent among the yield zones, ranging from 8.5 and 9.5 t ha^−1^ (Figure 6), indicating only minor differentiation among the yield zones. Across the yield zones, the UA fertilization system resulted in slightly higher yields. Significant differences (*p* < 0.01) in yield were found only in the low-yield zone, where the UA fertilization system resulted in a 0.3 t ha^−1^ higher yield (Table 4). The measured yields exceeded the targeted yields in the low- and medium-yield zones by 1.0 to 1.8 t ha^−1^. The target yield in the high-yield zone was overestimated by 1.0 t ha^−1^ and was not achieved.

The results showed that both fertilization systems led to similar variability of N uptake, N balance, and N efficiency. In the high-yield zone, the UA fertilization system had a significantly higher plant N uptake, with a difference of 6 kg ha^−1^ (*p* < 0.05). The VRA fertilization system involved applying significantly less fertilizer (median: 38 kg ha^−1^), which had a significant impact on N balance and N efficiency. The N balances were significantly lower across all yield zones (high-yield zone: 30 kg ha^−1^; medium-yield zone: 25 kg ha^−1^, *p* < 0.01, respectively; low-yield zone: 28 kg ha^−1^, *p* < 0.001). The N efficiencies were significantly higher compared with the UA fertilization system (high-yield zone: 10%, *p* < 0.05; medium-yield zone: 15%, *p* < 0.01; low-yield zone: 10%, *p* < 0.001).

The results from field C1 clearly demonstrate the superior performance of the VRA fertilization system compared with the UA system. The VRA resulted in lower N input, with nearly equal yield and protein content, leading to significantly lower N balances and higher N efficiency.

### 3.4. Field C2

Figure S8 illustrates the yield potential map and the N application maps (GS 32 + GS 39) for field C2. The distribution of the VRA was partially aligned with the yield zones, and N rates were generally high at both GSs.

The plant stand in field C2 encountered considerable stress due to particularly wet growth and cold growing conditions during the spring and early summer. These conditions led to yellow discoloration of the vegetation, affecting the quality of the reflectance measurements. There was a substantial discrepancy between Nup_cur_ and Nup_opt_, resulting in very high N rates suggested by the algorithm. The VRA fertilization system determined that the plant N uptake was insufficient to reach the target yield; therefore, high application rates of N fertilizer rates were required. In this field, the fertilizer rates were not limited by GFO regulations when using the VRA fertilization system.

The corrected N rates (reduced N rates due to leaf discoloration) remained highly variable, ranging from 14 to 88 kg ha^−1^ at GS 32 and from 42 to 90 kg ha^−1^ at GS 39 (Table S18). The mean N application rate at GS 32 was 52 kg ha^−1^, while the N application rate for the UA fertilization system was 35 kg ha^−1^. The mean N rate at GS 39 was 70 kg ha^−1^ for both N application methods. Overall, the VRA fertilization system involved the application of 20 kg ha^−1^ (median) more mineral fertilizer than the UA fertilization system.

**Figure 7:**
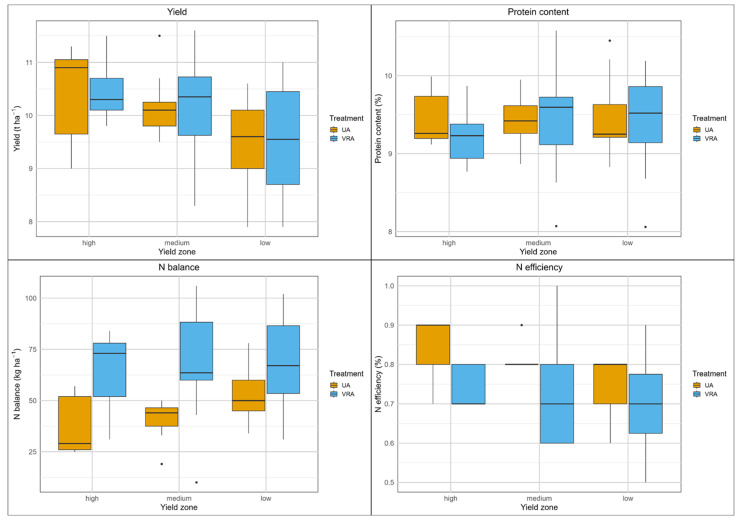
Boxplots of yield and N balance parameters, field C2, winter wheat, 2023.

The grain yield range of both fertilization systems was similar (7.9 and 11.6 t ha^−1^), with a median yield of 10.1 t ha^−1^ (Tables S19 and S20). The highest yield was recorded in the high-yield zone of the UA fertilization system (10.9 t ha^−1^), which was 0.6 t ha^−1^ higher than the corresponding yield under the VRA fertilization system (Table 5 and Figure 7). The lowest yields for both fertilization systems were observed in the low-yield zone (9.6 t ha^−1^). The observed differences in yield between the fertilization systems were minimal and thus not statistically significant. Furthermore, the measured yields in all yield zones exceeded the target yields. In the high-yield zone, the target yield was exceeded by 1.3 t ha^−1^, in the medium-yield zone by 1.9 t ha^−1^, and in the low-yield zone by 1.6 t ha^−1^.

Both fertilization systems resulted in similar ranges of N uptake, protein content, N balance, and N efficiency. The VRA fertilization system generally resulted in marginally higher values, with the exception of N efficiency. Protein contents were particularly low, with median values of 9.3% under the UA fertilization system and 9.4% under the VRA fertilization system. N balances were generally high, in particular under the VRA fertilization system (median: 68 kg ha^−1^). The median N balance under the UA fertilization system was 23 kg ha^−1^ lower. Across the yield zones, significantly higher N balances were observed for the VRA fertilization system, particularly in the high-yield zone (44 kg ha^−1^, *p* < 0.01), due to the high N rates. In the medium- and low-yield zones, the differences were 20 and 17 kg ha^−1^ (*p* < 0.05) respectively. The N efficiencies under the VRA fertilization system were 20% lower in the high-yield zone (*p* < 0.01) and 10% lower in the medium- and low-yield zones (*p* < 0.05, respectively).

Overall, the yields, N uptake, and protein contents of the two fertilization systems were at similar levels. However, the VRA system applied more N, which resulted in an increase in N balance of about 20 kg ha^−1^ and a 10% decrease in N efficiency, offering no advantages over the UA fertilization.

## 4. Discussion

### 4.1. Site Selection

These fertilizer trials were conducted at sites with different soil and management characteristics. The aim was to evaluate both fertilization systems (UA and VRA) under different site and management conditions, because variations in soil properties and management practices are major causes of site-specific differences in grain yield and N-balance parameters [1,6,42,70,71]. Furthermore, yield parameters are strongly affected by meteorological conditions. In years characterized by drought stress, the distinction between yield zones is more pronounced than in years with a more even distribution of precipitation, especially in critical phases of the wheat growing season [72,73]. Since the selected sites were relatively close to each other (maximum distance 150 km), the climate conditions (30-year average) were not very different (Tables S1–S3)). However, the annual weather conditions varied considerably—in the very wet year 2021, precipitation at Site A was 914 mm, while in the dry year 2022, it was only 692 mm at Site B and 642 mm at Site C. Therefore, the fertilization systems were tested in practice under different conditions.

Due to the technical and personnel requirements and the high costs associated with this method, only a few fields could be investigated. The methodology should therefore be further developed so that significantly more fields can be efficiently investigated. This can be achieved by replacing the labor-intensive yield determination using plot combine harvesters by independent digital systems such as drones equipped with hyperspectral cameras. In addition, analyses should include further sites that have completely different conditions from those in the current study’s fields in Bavaria, e.g., very large fields in northeastern Germany ranging in size from 50 to over 100 ha, small-scale extremely variable soil conditions, and pronounced periods of drought and heat stress due to local climatic conditions [72,73]. The possibility that the advantages of the VRA fertilization method become more pronounced under these conditions cannot be excluded. However, the transfer of the VRA fertilization method is not trivial, since, for example, the DIMA factor (see Section 2.3.2) has to be validated under significantly different soil conditions.

### 4.2. Discussion of Methods

The conduction of strip trials is a highly labor-intensive process that requires large areas for implementation. However, they accurately reflect the conditions of agricultural practice and are therefore suitable for a practical analysis of the efficiency of sensor-based site-specific N fertilization compared with uniform field fertilization. The main advantage of strip trials over randomized plot trials is their better representation of the spatial variability of arable fields. In contrast, plot trials for testing fertilization systems [53] are usually conducted on homogeneous areas, which limits their ability to accurately reflect real conditions in agricultural fields.

Sensor-based site-specific N fertilization based on the online + map-overlay method is a sophisticated process that is very complex and challenging to use. The map indicates the yield potential and provides an initial estimate of the N rates for each yield zone, based on the target yield [29]. Subsequently, real-time sensor measurements refine the initial estimation in accordance with the plants’ current development [27,28]. Therefore, one of the most important prerequisites for site-specific fertilization is the precise determination of yield zones. There are several methods for the delineation of site-specific yield zones, including historical yield maps, yield determination by combine harvesters, and sensor- or satellite-based yield estimation. The determination of yield zones based on multi-year satellite images represents a suitable method that can be carried out with limited effort for almost all arable fields. The multi-year yield patterns reflect the influence of relatively stable soil parameters, e.g., soil texture, water-storage capacity, and organic carbon content [2,31].

However, for site-specific fertilization of winter wheat, it is not sufficient to determine yield zones and relative biomass potentials [31]; rather, as in the field trials conducted in the present study, absolute target yields must be assigned to the yield zones.

In the present study, the low-, medium- and high-yield zones were assigned grain yields (e.g., 7 t ha^−1^, 8 t ha^−1^, 9 t ha^−1^) in consideration of the average yields measured at the weighbridge. Since the algorithm of the online + map-overlay fertilizer application method is highly dependent on the yield potential of each yield zone, inaccurate estimations of the yield potential would negatively impact the accuracy of the results. Consequently, the estimated N rates according to the calculation may be either too high or too low for each yield zone. To address this deficiency, it is crucial to integrate a highly reliable method for accurate derivation of site-specific yields in t ha^−1^. This will ensure consistent calculation of appropriate fertilizer rates using the algorithm.

In this study, the GFO official fertilization system was used as a reference system for the uniform application. The GFO regulations set legally prescribed upper limits for N fertilization and recommend specific application rates for different dates (at the beginning of vegetation in spring, at GS 32, and GS 39).

The GFO fertilization system was correctly applied at the study sites, resulting in high yields and high N efficiency. A number of studies have demonstrated the effectiveness of the GFO system for determining N fertilizer application requirements. In particular, yield potential was largely achieved and low N balance values simultaneously obtained at sites with low mineralization potential and in arable farming systems without organic fertilization [53,74]. In farming systems with intensive organic fertilization (with slurry or biogas digestate), the GFO system has not always been entirely accurate in determining optimal rates of fertilizer application [52]. In general, when comparing the UA and VRA fertilization systems, the more effectively the GFO (UA) fertilization system is implemented, the more difficult it is for the VRA fertilization method to achieve better results. In field A1 in the present study, the N fertilizer requirement determined using the VRA fertilization system was limited by the upper limit set by the GFO, while this limit was not implemented in fields B1, C1, or C2. Maintaining the GFO upper limit resulted in a reduction in fertilizer application under the VRA fertilization system in some areas of the field, depending on the yield zone and the development of the crop stand. Under this system, it is also necessary to determine whether the GFO fertilization limit applies to each sub-area or only to the average of the arable field. The latter allows greater flexibility in fine-tuning the application of fertilizer.

### 4.3. Discussion of Results

The results from field A1 demonstrated the superiority of the VRA fertilization system compared with the UA fertilization system, as mineral N input was reduced without negative impact on yield. This resulted in reduced N balance and improved N efficiency.

These findings align with results of previous studies [14,52]. Prücklmaier demonstrated through both plot trials and field-scale trials that the VRA fertilization system required significantly reduced N application rates compared with the UA fertilization system. In some cases, additional N application at growth stages GS 32 or GS 39 could be omitted completely without adverse effects on yield. However, these studies were conducted on fields with high potential for N mineralization due to long-term intensive application of slurry.

Such fields were not included in the current investigations. There is a strong likelihood that the potential for mineral N savings is higher for fields with intensive organic fertilization than fields without organic fertilization. However, further research is needed to confirm this.

Field B1 contained two distinctive soil types, a common condition found in the study region. This raises the fundamental question of whether it is more appropriate to divide such fields based on soil types or to leave them as they are and manage them with site-specific techniques. In field B1, the VRA fertilization system led to higher N applications than the UA fertilization system, which resulted in significantly higher protein content. Consequently, N efficiency decreased and N balances increased significantly. However, the absolute level of N balance achieved with the VRA fertilization system (22 to 34 kg ha^−1^) in the yield zones remained within a moderate range [75,76]. Therefore, under these conditions, VRA fertilization would not contribute to positive environmental effects. A potential solution could be to set an upper limit for N application according to the GFO.

The VRA fertilization system demonstrated superior performance in field C1, significantly reducing mineral N rates while maintaining yield and protein content. This resulted in enhanced N efficiency and reduced N balance, leading to a balanced N budget. However, there were only minor yield differences between the yield zones across the study year. The relative biomass potential method [31] revealed significant yield differences based on historical satellite data. However, these differences were not reflected in the results. Across the study year, the variability in yield from this field was minimal. This indicates that in years with favorable weather conditions, the impact of yield zones was less significant, resulting in consistently high yields in all yield zones. At field C2, higher N balances and lower N efficiencies were obtained with the VRA fertilization system, due to the high rate of N application required by the VRA system. The N application rates in this field had to be corrected manually at both growth stages. Nevertheless, these corrections were insufficient. At GS 33, the plant stand encountered considerable stress due to particularly wet and cold growing conditions in April. Water-saturated soils have a negative effect on roots, as heavy rainfall induces transient hypoxia until soil water saturation levels normalize [77]. In this case, the water saturation was still notable during harvest (20 July). These hypoxic conditions can cause significant damage to crop production by adversely affecting shoot physiology [78]. Plant stress at GS 33 was indicated by yellow discoloration of the leaves. This affected the quality of the reflectance measurements. There was a considerable discrepancy between N_cur_ and N_opt_; accordingly, the algorithm suggested very high N application rates. Nevertheless, the N application rates were reduced at both growth stages. Considering the results, the adjustments of these rates were insufficient. 

## 5. Conclusions and Outlook

Overall, the VRA fertilization system demonstrated encouraging potential, functioning as intended. However, it still requires significant input from experts to ensure optimal calibration. To enhance accessibility and efficiency, further adjustments are necessary to ensure that the VRA fertilization system consistently results in strong outcomes and enhances N efficiency under the variable conditions typically encountered in agricultural practice.

A primary aspect of this study is the implementation of an upper limit to N application rates in accordance with the guidelines set forth by the GFO to prevent over-fertilization. Further studies including field experiments are required to refine and validate the DIMA factor and N-uptake models and to adapt the models to site-specific conditions, enabling more precise application. Another crucial objective is to optimize the sensor system through the application of AI. The integration of AI into the algorithms can make it possible to reliably detect the N nutrition status of crops, even in complex situations such as plant diseases or physiological leaf coloring. Finally, there is a need to develop a universally applicable algorithm for deriving yield zones and assigning absolute yields without relying on expert estimations. This would make the approach more user-friendly and widely accessible for farmers and practitioners.

In ongoing research, this method will be tested on larger fields (up to 50 ha) and at sites with greater differences in soil and climate conditions, including dry sites, sandy soils, and fields with greater within-field heterogeneity. Furthermore, AI implementations connected with current climatic data sources will be integrated into the system to automatically adapt the algorithms to the current site conditions, ensuring more accurate and dynamic responses to environmental variability.

## Figures and Tables

**Table 1:** Study fields.

Experimental Site	Research Station Dürnast	Research Station Roggenstein	Commercial Farm Burghausen	Commercial Farm Burghausen
Field	A1	B1	C1	C2
Size (ha)	4.7	27.0	7.5	9.5
Region	30 km north of Munich	20 km west of Munich	80 km east of Munich	80 km east of Munich
Soil texture	Loam	Sandy loam	Silty loam	Silty loam
Soil type	Cambisol	Cambisol/gley	Cambisol	Cambisol
SOC (% DM)	1.4 (1.2–2.1)	3.2 (2.3–8.7) ^a^	1.3 (1.1–2.1)	1.5 (1.4–1.8)
TN (% DM)	0.16 (0.11–0.23)	0.22 (0.10–0.34)	0.15 (0.12–0.22)	0.15 (0.13–0.19)
pH (CaCl_2_)	7.0 (6.2–7.3)	6.5 (6.1–7.0)	6.5 (6.0–7.2)	6.5 (5.9–7.0)
P_CAL_ (mg (100 g)^−1^) ^b^	5.6 (3.4–12.4)	4.8 (4.0–5.4)	7.6 (3.1–31.7)	4.1 (2.2–7.6)
K_CAL_ (mg (100 g)^−1^) ^c^	13.9 (9.1–26.1)	17 (15.0–20.0)	12.6 (7.0–25.3)	7.6 (2.9–15.0)
Height	479 (472–487)	517 (515–519)	482 (480–484)	474 (470–478)
Farming system	Arable farming	Arable farming	Mixed farming	Mixed farming
Coordinates	48°26′4″ N 11°44′16″ E	48°10′47″ N 11°18’50″ E	48°7′51″ N 12°44′5″ E	48°7′58″ N 12°44′22″ E

^a^ This field contained two soil types (Cambisol, sandy loam (68% of the area) and gley, (32% of the area)), characteristic of the study site and the soils at the experimental station. According to GFO [59], a deduction of 20 kg ha^−1^ was made when determining N fertilizer requirements for soils with a soil organic matter content of >4% (as in this sub-plot with gley soils). ^b^ P_CAL_ contents were primarily within the range of optimal soil nutrient levels, indicating that P_CAL_ levels across the field were not considered yield-limiting. ^c^ K_CAL_ contents were primarily within the range of optimal soil nutrient levels, indicating that K_CAL_ levels across the field were not considered yield-limiting.

**Table 2:** Yield and N balance parameters (median) of the yield zones, winter wheat, field A1, 2021.

Yield Zone	Fertilization System	Target Yield	N	Yield	Protein Content	Plant N Uptake ^a^	N Fertilization ^b^	N Balance	N Efficiency
		(t ha^−1^)		(t ha^−1^)	(%)	(kg ha^−1^)	(kg ha^−1^)	(kg ha^−1^)	
High-yield	UA	8.0	10	10.4	12.4	230	155	−75	1.48
zone	VRA	9.0	19	10.4	12.2	230	160	−70	1.46
Medium-yield	UA	8.0	12	10.1 *	10.5	198	155	−43 *	1.28 **
zone	VRA	8.0	33	9.8 *	10.9	196	134	−64 *	1.46 **
Low-yield	UA	8.0	11	9.7	10.3	191	155	−36 **	1.23 **
zone	VRA	7.0	18	9.5	10.7	188	135	−53 **	1.38 **
Median	UA			34	10.1	10.6	198	155	1.28
Median	VRA			70	9.8	11.0	201	140	1.43

UA: uniform fertilizer application. VRA: variable-rate fertilizer application. ^a^ N uptake in grain and straw. ^b^ VRA limited by GFO (at field level). * Significance levels: * *p* < 0.05, ** *p* < 0.01.

**Table 3:** Yield and N balance parameters (median) of the yield zones, winter wheat, field B1, 2022.

Yield Zone	Fertilization System	Target Yield	N	Yield	Protein Content	Plant N Uptake ^a^	N Fertilization ^b^	N Balance	N Efficiency
		(t ha^−1^)		(t ha^−1^)	(%)	(kg ha^−1^)	(kg ha^−1^)	(kg ha^−1^)	
High-yield	UA	8.0	30	8.2	9.5 ***	142 *	155	13 ***	0.92 ***
zone	VRA	9.0	49	8.4	9.8 ***	150 *	177	28 ***	0.84 ***
Medium-yield	UA	8.0	39	8.6	9.8 ***	153 ***	155	2 ***	0.99 ***
zone	VRA	8.0	41	8.6	10.4 ***	159 ***	192	34 ***	0.82 ***
Low-yield	UA	8.0	31	8.8	10.8	170	155	−15 ***	1.10 ***
zone	VRA	7.0	32	8.7	11.3	177	198	22 ***	0.89 ***
Median	UA		100	8.5	9.9	153	155	2	0.99
Median	VRA		122	8.5	10.2	155	189	28	0.85

UA: uniform fertilizer application. VRA: variable-rate fertilizer application. ^a^ N uptake in grain and straw. ^b^ VRA limited by GFO (at field level). * Significance levels: * *p* < 0.05, ** *p* < 0.01, *** *p* < 0.001.

**Table 4:** Yield and N balance parameters (median) of the yield zones, winter wheat, field C1, 2022.

Yield Zone	Fertilization System	Target Yield	N	Yield	Protein Content	Plant N Uptake ^a^	N Fertilization ^b^	N Balance	N Efficiency
		(t ha^−1^)		(t ha^−1^)	(%)	(kg ha^−1^)	(kg ha^−1^)	(kg ha^−1^)	
High-yield	UA	9.0	14	9.3	11.3	189 *	206	23 **	0.90 *
Zone	VRA	10.0	13	9.0	10.7	183 *	178	−7 **	1.00 *
Medium-yield	UA	9.0	8	9.3	11.7	189	206	17 **	0.90 **
Zone	VRA	8.0	10	9.0	10.7	169	163	−8 **	1.05 **
Low-yield	UA	9.0	11	9.1 **	11.2	179	206	27 ***	0.90 ***
Zone	VRA	7.0	9	8.8 **	10.5	164	164	−1 ***	1.00 ***
Median	UA			9.2	11.3	182	206	24	0.90
Median	VRA			9.0	11.0	172	168	−7	1.00

UA: uniform fertilizer application. VRA: variable-rate fertilizer application. ^a^ N uptake in grain and straw. ^b^ VRA limited by GFO (at field level). * Significance levels: * *p* < 0.05, ** *p* < 0.01, *** *p* < 0.001.

**Table 5:** Yield and N balance parameters (median) of the yield zones, winter wheat, field C2, 2022.

Yield Zone	Fertiliztion System	Target Yield	N	Yield	Protein Content	Plant N Uptake ^a^	N Fertilization ^b^	N Balance	N Efficency
		(t ha^−1^)		(t ha^−1^)	(%)	(kg ha^−1^)	(kg ha^−1^)	(kg ha^−1^)	
High-yield	UA	8.5	7	10.9	9.3	187	216	29 **	0.90 **
zone	VRA	9.0	9	10.3	9.2	178	253	73 **	0.70 **
Medium-yield	UA	8.5	15	10.1	10.1	172	216	44 *	0.80 *
zone	VRA	8.5	12	10.4	10.4	181	245	64 *	0.70 *
Low-yield	UA	8.5	13	9.6	9.3	166	216	50 *	0.80
zone	VRA	8.0	10	9.6	9.5	163	228	67 *	0.70
Median	UA		35	10.1	9.3	170	216	46	0.80
Median	VRA		31	10.1	9.4	177	241	67	0.70

UA: uniform fertilizer application. VRA: variable-rate fertilizer application. ^a^ N uptake in grain and straw. ^b^ VRA limited by GFO (at field level). * Significance levels: * *p* < 0.05, ** *p* < 0.01.

## Data Availability

The data supporting the conclusions of this article will be made available by the authors on request.

## Supplementary Materials

The following supporting information can be downloaded at https://www.mdpi.com/article/10.3390/s25030795/s1: Table S1: Mean temperature and precipitation at site Aa; Figure S1: Distribution of soil types at field B1 according to the German soil appraisal; Table S2: Mean temperature and precipitation at site Ba; Table S3: Mean temperature and precipitation at Site Ca; Table S4: Farm management, field A1; Table S5: Farm management, field B1; Table S6. Farm management, field C1; Table S7: Farm management, field C2; Figure S2: Compact Spec tractor-mounted multispectral sensor system; Figure S3: Recording of tramlines in field C1 (a) and plot design according to the yield potential map (red as low-yield zone, yellow as medium-yield zone, green as high-yield zone (b); Figure S4: Georeferenced spectral data points in field C1 (a), average REIP of VRA plots at growth stage GS 32 (b); Figure S5: Plot layout; Table S8: Descriptive methods for plant data; Table S9: Descriptive statistics of N fertilization, field A1, winter wheat, 2021; Table S10: Descriptive statistics of N fertilization, field A1, winter wheat, 2021 (UA); Table S11: Descriptive statistics of the yield parameters, field A1, winter wheat, 2022 (VRA); Figure S6: Yield potential map (a) and N fertilizer application maps, determined N fertilizer requirements, and N fertilizer application rates at GS 31 (b) and GS 39 (c), field B1 (27 ha), winter wheat, 2022; Table S12: Descriptive statistics of N fertilization, field B1, winter wheat, 2022; Table S13: Descriptive statistics of the yield parameters, field B1, winter wheat, 2022 (UA); Table S14: Descriptive statistics of the yield parameters, field B1, winter wheat, 2022 (VRA); Figure S7: Yield potential map (a) and N fertilizer application maps, determined N fertilizer requirements, and N fertilizer application rates at GS 32 (b) and GS 43 (c), field C1 (7.5 ha), winter wheat, 2022; Table S15: Descriptive statistics of N fertilization, field C1, winter wheat, 2022; Table S16: Descriptive statistics of the yield parameters, field C1, winter wheat, 2022 (UA); Table S17: Descriptive statistics of the yield parameters, field C1, winter wheat, 2022 (VRA); Figure S8: Yield potential map (a) and N fertilizer application maps, determined N fertilizer requirements, and N fertilizer application rates at GS 33 (b) and GS 39 (c), field C2 (9.5 ha), winter wheat, 2023; Table S18: Descriptive statistics of N fertilization, field C2, winter wheat, 2023; Table S19: Descriptive statistics of the yield parameters, field C2, winter wheat, 2022 (UA); Table S20: Descriptive statistics of the yield parameters, field C2, winter wheat, 2022 (VRA). References [31,67,79] are cited in the Supplementary Materials.
