# Supplementary material for: Effects of Sensor-Based, Site-Specific Nitrogen Fertilizer Application on Crop Yield, Nitrogen Balance, and Nitrogen Efficiency
Source: Sensors (Basel). 2025 Jan 28;25(3):795. doi: 10.3390/s25030795 (PMC11820920; doi:10.3390/s25030795)
Supplement: Supplementary file 1 [file sensors-25-00795-s001.zip › sensors-3355037-supplementary.pdf]

Article

Effects of Sensor-Based, Site-Specific Nitrogen Fertilizer Application on Crop Yield, Nitrogen Balance, and Nitrogen Efficiency

Ludwig Hagn [1,*], Martin Mittermayer [1], Andreas Kern [2], Stefan Kimmelmann [2], Franz-Xaver Maidl [1] and Kurt-Jürgen Hülsbergen [1]

[1] Chair of Organic Agriculture and Agronomy, Technische Universität München, Liesel-Beckmann-Straße 2, 85354 Freising, Germany; martin.mittermayer@tum.de (M.M.); maidl@tum.de (F.X.M.); kurt.juergen.huelsbergen@tum.de (K.-J.H.)

[2] Field Crops Unit, Plant Technology Center, Technische Universität München, Dürnast 5, 85354 Freising, Germany; andreas.kern@tum.de (A.K.); stefan.kimmelmann@tum.de (S.K.)

* Correspondence: ludwig.hagn@tum.de

Table S1. Mean temperature and precipitation at site A[a]

Parameter	unit	Jan	Feb	Mar	Apr	May	Jun	Jul	Aug	Sep	Oct	Nov	Dec	Year
**1991-2020**														
Temperature x̄	°C	-0.7	0.6	4.3	9.0	13.3	16.9	18.1.	17.8	13.3	8.9	3.7	0.5	8.8
Precipitation ∑	mm	50.4	39.2	51.6	49.0	91.5	100.1	102.1	94.5	66.3	61.9	53.5	53.2	813.1
**2021**														
Temperature x̄	°C	-0.7	2.3	4.1	6.3	10.5	18.8	17.9	16.4	14.5	8.0	2.8	2.0	8.6
Precipitation ∑	mm	53.7	40.2	36.6	29.0	161.0	131.0	114.8	166.7	35.9	17.7	36.9	90.9	914.4

[a] Freising weather station (1 km north of the study fields)

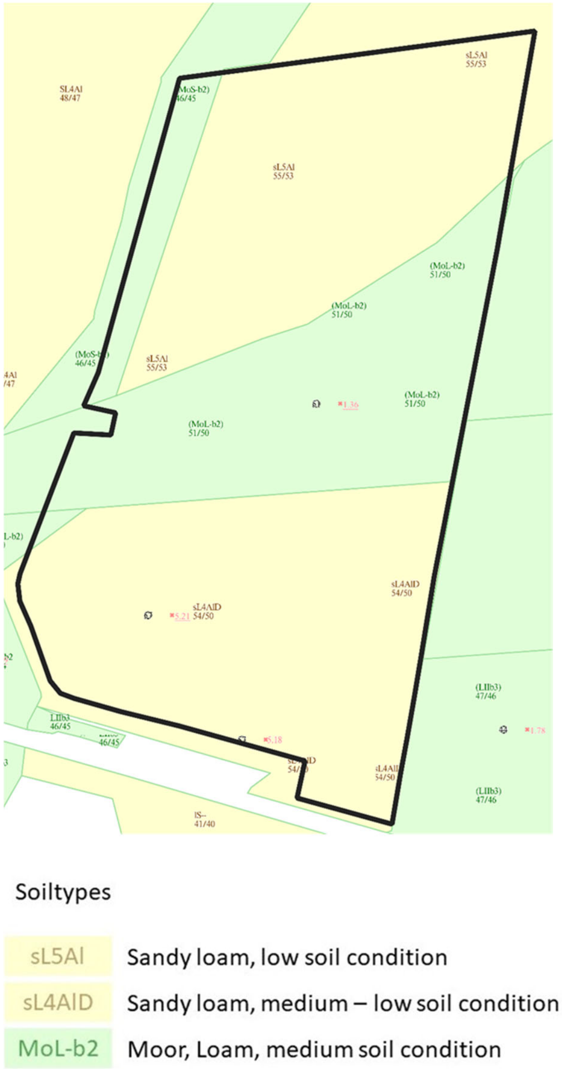

Figure S1. Distribution of soil types at field B1 according to the German soil appraisal (yellow: mineral soil (Cambisol); green: organic soil (Gley))

Table S2. Mean temperature and precipitation at site B[a]

Parameter	unit	Jan	Feb	Mar	Apr	May	Jun	Jul	Aug	Sep	Oct	Nov	Dec	Year
**1991-2020**														
Temperature x̄	°C	-0.7	0.6	4.3	9.0	13.3	16.9	18.1.	17.8	13.3	8.9	3.7	0.5	8.8
Precipitation ∑	mm	50.4	39.2	51.6	49.0	91.5	100.1	102.1	94.5	66.3	61.9	53.5	53.2	813.1
**2021**														
Temperature x̄	°C	-0.7	2.3	4.1	6.3	10.5	18.8	17.9	16.4	14.5	8.0	2.8	2.0	8.6
Precipitation ∑	mm	53.7	40.2	36.6	29.0	161.0	131.0	114.8	166.7	35.9	17.7	36.9	90.9	914.4

[a] Maisach-Galgen weather station (10 km west of the study fields)

Table S3. Mean temperature and precipitation at site C[a]

Parameter	unit	Jan	Feb	Mar	Apr	May	Jun	Jul	Aug	Sep	Oct	Nov	Dec	Year
**1991-2020**														
Temperature x̄	°C	-0.7	0.6	4.3	9.0	13.3	16.9	18.1.	17.8	13.3	8.9	3.7	0.5	8.8
Precipitation ∑	mm	50.4	39.2	51.6	49.0	91.5	100.1	102.1	94.5	66.3	61.9	53.5	53.2	813.1
**2021**														
Temperature x̄	°C	-0.7	2.3	4.1	6.3	10.5	18.8	17.9	16.4	14.5	8.0	2.8	2.0	8.6
Precipitation ∑	mm	53.7	40.2	36.6	29.0	161.0	131.0	114.8	166.7	35.9	17.7	36.9	90.9	914.4

[a] Frieding weather station (15 km north of the study field)

Table S4. Farm management, field A1

**Field A1**	**unit**	**Jan**	**Feb**	**Mar**
Study year				2020/2021
Previous crop		Rape seed		
Crop		Winter wheat		
Variety		Apostel		
Seed rate	grains m^{-2}	320		
NR_{sy}	N, kg ha^{-1}	230[a]		
FR_{sd}	N, kg ha^{-1}	155 [a]		
TY	t ha^{-1}	8.0[a]		
SMN_{vs} (0-60 cm)	N, kg ha^{-1}	65[a]	Soil sampling	08.02.2021
Target protein content	%	13		
**Treatment**		**Amount**	**Fertilizer, plant protection product**	
Tillage (cultivator)				09.10.2020
Sowing (rotary harrow, seed drill)				10.10.2020
First application of mineral N fertilizer	N, kg ha^{-1}	50[c]	AS	19.03.2021
Second application of mineral N fertilizer	N, kg ha^{-1}	44[b]/70[c]	CAN	11.05.2021
Third Application of mineral N fertilizer	N, kg ha^{-1}	49[b]/35[c]	CAN	04.06.2021
Total N fertilizer	N, kg ha^{-1}	142[b]/155[c]		
Application of crop protection measures	l ha^{-1}	2.0 + 0.2	CTU 700 + Sempra	30.10.2021
Application of crop protection measures	l ha^{-1}	0.9 + 0.4	Input Classic + Prodax	29.04.2021
Application of crop protection measures	l ha^{-1}	1.0 + 0.33	Elatus Era + Sympara	21.05.2021
Harvest[d]				07.08.2021

[a] required parameters for the determination of the N fertilizer requirement according to the German Fertilizer Ordinance (2017): NRsy (N requirement at standard yield), FRsd (Fertilizer N requirement after surcharges and deductions), TY (Target yield), SMNvs (Soil mineral N stock at vegetation start); [b] Mean amount of applied N (VRA); [c] Mean amount of applied N (UA).

Table S5. Farm management, field B1

**Field B1**	**unit**	**Jan**	**Feb**	**Mar**
Study year				2021/2022
Previous crop		corn		
Crop		Winter wheat		
Variety		Asory		
Seed rate	grains m^{-2}	300		
NR_{sy}	N, kg ha^{-1}	230[a]		
FR_{sd}	N, kg ha^{-1}	155 [a]		
TY	t ha^{-1}	8.0[a]		
SMN_{vs} (0-60 cm)	N, kg ha^{-1}	65[a]	Soil sampling	13.02.2022
Target protein content	%	13		
**Treatment**		**Amount**	**Fertilizer, plant protection product**	
Tillage (cultivator)				08.10.2021
Sowing (rotary harrow, seed drill)				11.10.2021
Application of organic fertilizer	N, kg ha^{-1}			
First application of mineral N fertilizer	N, kg ha^{-1}	18[c] + 52[c]	DAP + AS	04.03.2021
Second application of mineral N fertilizer	N, kg ha^{-1}	54[b]/45[c]	CAN	04.05.2021
Third application of mineral N fertilizer	N, kg ha^{-1}	65[b]/40[c]	CAN	22.05.2021
Total N fertilization	N, kg ha^{-1}	189[b]/155[c]		
Application of crop protection measures	l ha^{-1}	2.0	Trinity	30.10.2021
Application of crop protection measures	l ha^{-1}	0.8 + 5.0	CCC + Bittersalz	12.04.2022
Application of crop protection measures	l ha^{-1}	0.8 + 0.4	Proline + Prodax	03.05.2022
Application of crop protection measures	l ha^{-1}	1.0 + 0.075	Elatus Era + Karate Zeon	30.05.2022
Application of crop protection measures	l ha^{-1}	1.5	Stamina XZ	31.05.2022
Application of crop protection measures	l ha^{-1}	1.0	Prosaro	08.06.2022
Harvest[d]				03.08.2022

[a] required parameters for the determination of the N fertilizer requirement according to the German Fertilizer Ordinance (2017): NRsy (N requirement at standard yield), FRsd (Fertilizer N requirement after surcharges and deductions), TY (Target yield), SMNvs (Soil mineral N stock at vegetation start); [b] Mean amount of applied N (VRA); [c] Mean amount of applied N (UA).

Table S6. Farm management, field C1

**Field C2**	**unit**	**Jan**	**Feb**	**Mar**
Study year				2021/2022
Previous crop		Silage corn		
Crop		Winter wheat		
Variety		Spontan		
Seed rate	grains m^{-2}	320		
NR_{sy}	N, kg ha^{-1}	240[a]		
FR_{sd}	N, kg ha^{-1}	175 [a]		
TY	t ha^{-1}	9.0[a]		
SMN_{vs} (0-60 cm)	N, kg ha^{-1}	35[a]	Soil sampling	06.02.2022
Target protein content	%	13		
**Treatment**		**Amount**	**Fertilizer, plant protection product**	
Tillage (plow)				09.10.2021
Sowing (rotary harrow, seed drill)				10.10.2021
Application of organic fertilizer	N, kg ha^{-1}	96[c] (63[d])	Biogas slurry + Vizura stabilizer	10.03.2022
First application of mineral N fertilizer	N, kg ha^{-1}	30[c]	NPK 14/10/20	09.03.2022
Second application of mineral N fertilizer	N, kg ha^{-1}	36[b]/50[c]	CAN	04.05.2022
Third application of mineral N fertilizer	N, kg ha^{-1}	5[b]/30[c]	CAN	20.05.2022
Total N fertilization	N, kg ha^{-1}	134[b]/173[c]		
Total N fertilization (incl. organic N)	N, kg ha^{-1}	167[b]/206[c]		
Application of crop protection measures	l ha^{-1}	2.0 + 0.2	CTU 700 + Sempra	30.10.2021
Application of crop protection measures	l ha^{-1}	0.9 + 0.4	Input Classic + Prodax	29.04.2022
Application of crop protection measures	l ha^{-1}	1.0 + 0.33	Elatus Era + Sympara	21.05.2022
Harvest[d]				19.07.2022

[a] required parameters for the determination of the N fertilizer requirement according to the German Fertilizer Ordinance (2017): NRsy (N requirement at standard yield), FRsd (Fertilizer N requirement after surcharges and deductions), TY (Target yield), SMNvs (Soil mineral N stock at vegetation start); [b] Mean amount of applied N (VRA); [c] Mean amount of applied N (UA)

Table S7. Farm management, field C2

**Field C2**	**unit**	**Jan**	**Feb**	**Mar**
Study year				2022/2023
Previous crop		Silage corn		
Crop		Winter wheat		
Variety		Apostel		
Seed rate	grains m^{-2}	320		
NR_{sy}	N, kg ha^{-1}	235[a]		
FR_{sd}	N, kg ha^{-1}	185[a]		
TY	t ha^{-1}	8.5[a]		
SMN_{vs} (0-60 cm)	N, kg ha^{-1}	29[a]	Soil sampling	11.02.2023
Target protein content	%	13		
**Treatment**		**Amount**	**Fertilizer, plant protection product**	
Tillage (plow)				05.10.2022
Sowing (rotary harrow, seed drill)				06.10.2022
Application of organic fertilizer	N, kg ha^{-1}	76 (44[d])[c]	cattle slurry	10.03.2023
First application of mineral N fertilizer	N, kg ha^{-1}	36	Sulfan	09.03.2022
Second application of mineral N fertilizer	N, kg ha^{-1}	52[b]/35[c]	Sulfan	04.05.2022
Third application of mineral N fertilizer	N, kg ha^{-1}	70[b]/70[c]	CAN	20.05.2022
Total N fertilizer	N, kg ha^{-1}	202[b]/185[c]		
Total N fertilizer (incl. organic N)	N, kg ha^{-1}	234[b]/217[c]		
Application of crop protection measures	l ha^{-1}	2 + 0.2	CTU 700 + Sempra	30.10.2021
Application of crop protection measures	l ha^{-1}	0.9 + 0.4	Input Classic + Prodax	29.04.2022
Application of crop protection measures	l ha^{-1}	1.0 + 0.33	Elatus Era + Sympara	21.05.2022
Harvest[d]				20.07.2023

[a] required parameters for the determination of the N fertilizer requirement according to the German Fertilizer Ordinance (2017): NRsy (N requirement at standard yield), FRsd (Fertilizer N requirement after surcharges and deductions), TY (Target yield), SMNvs (Soil mineral N stock at vegetation start); [b] Mean amount of applied N (VRA); [c] Mean amount of applied N (UA).

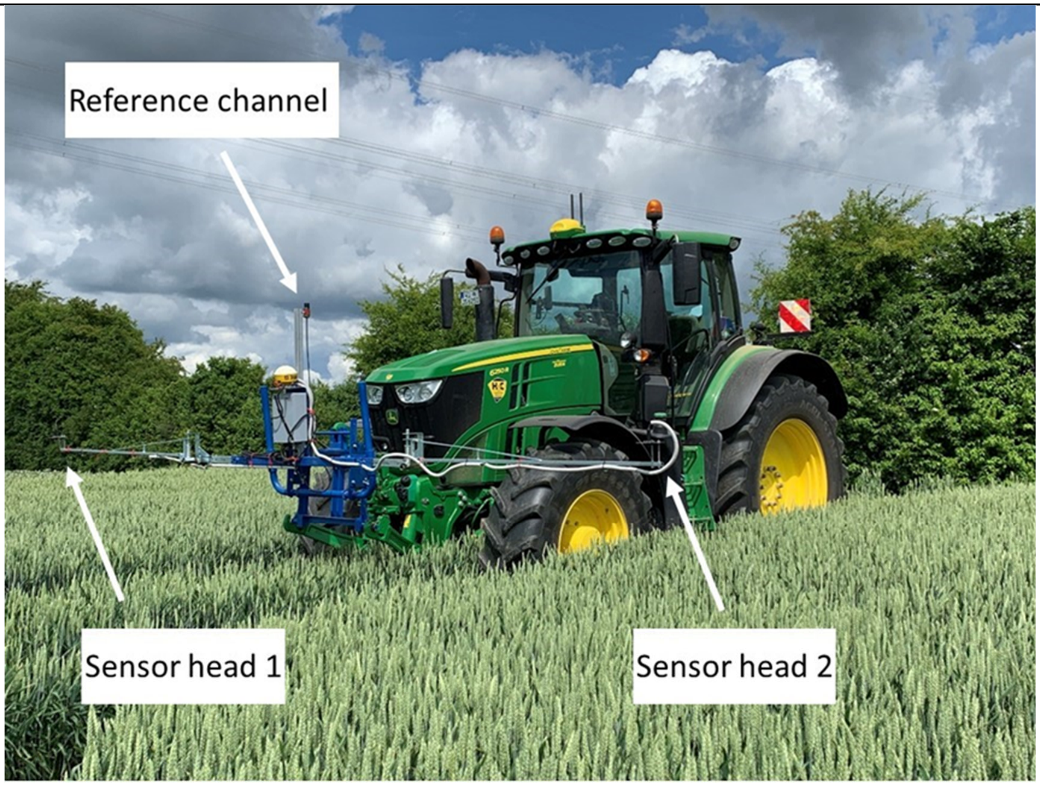

Figure S2. Compact Spec tractor-mounted multispectral sensor system (tec5 2021)

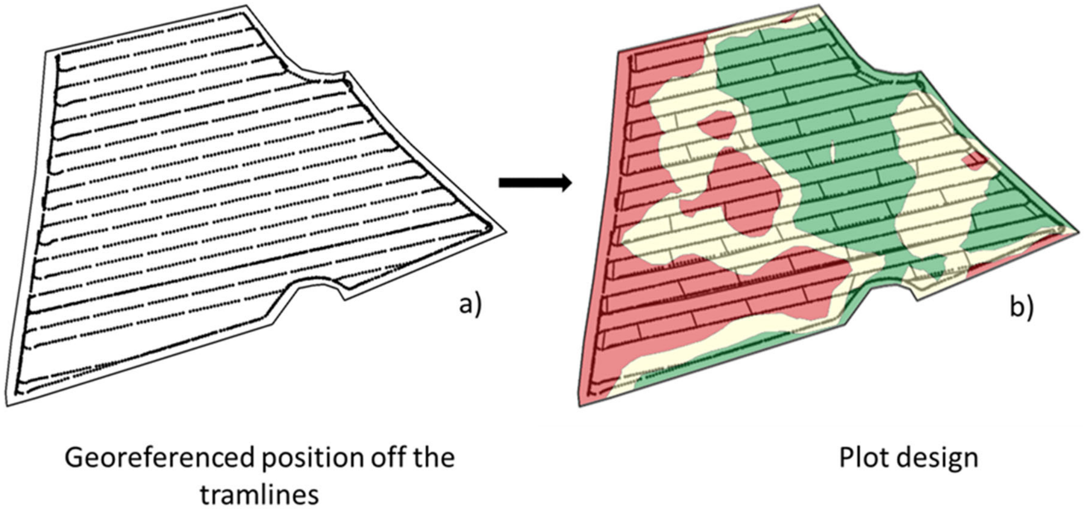

Figure S3. Recording of tramlines of field C1 (a) and plot design according to the yield potential map (red as low-yield zone, yellow as medium-yield zone, green as high-yield zone (b).

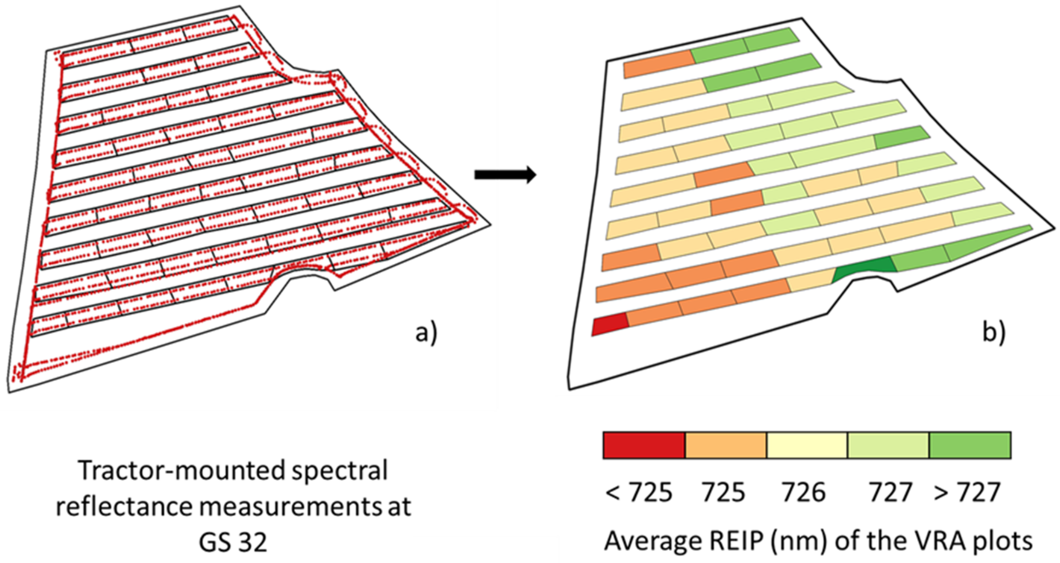

Figure S4. Georeferenced spectral data points at field C1 (a); average REIP of VRA plots at growth stage GS 32 (b).

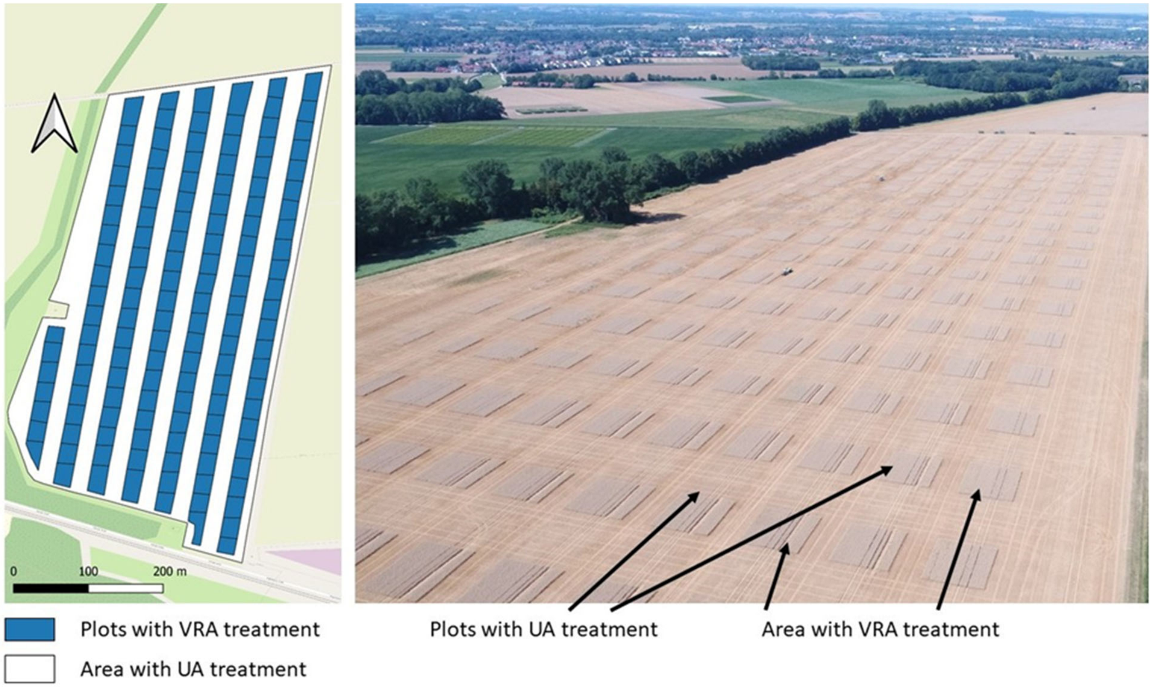

Figure S5. Plot layout

Table S8. Descriptive methods for plant data

**Plant parameters**				
**Variable**	**Unit**	**Data source**	**Method**	**Literature**
a) based on combine harvester				
Grain yield[a]	t ha^{-1}	Combine harvester	Weighing cells	[1]
Grain N content	%	Biomass samples from combine harvester	Dumas combustion method	[2]
Grain N uptake	kg ha^{-1}	Biomass samples from combine harvester	Dumas combustion method	[2]
b) based on satellite data				
Rel. BMP	%	Satellite data	Multi-year NDVI raster/mean multi-year NDVI field*100	[3]
c) based on tractor-mounted reflectance measurements				
Vegetation index	REIP	Tractor mounted sensor[b]	Reflectance measurements	[4]
a) based on combine harvester				
Grain yield[a]	t ha^{-1}	Combine harvester	Weighing cells	[1]
Grain N content	%	Biomass samples from combine harvester	Dumas combustion method	[2]
Grain N uptake	kg ha^{-1}	Biomass samples from combine harvester	Dumas combustion method	[2]
b) based on satellite data				
Rel. BMP	%	Satellite data	Multi-year NDVI raster/mean multi-year NDVI field*100	[3]
c) based on tractor-mounted reflectance measurements				
Vegetation index	REIP	Tractor mounted sensor[b]	Reflectance measurements	[4]

[a] Grain yield (14 % moisture content),

[b] tractor-mounted reflectance measurements sensor (350-1140 nm)

Table S9. Descriptive statistics of N fertilization, field A1, winter wheat, 2021

Parameter	Fertilization system	n	Year	Unit	Mean	Median	Min	Max	SD
Fertilization EC 32	VRA	70	2021	kg ha^{-1}	44	43	22	70	11.32
Fertilization EC 32	UA	34	2021	kg ha^{-1}	70				
Fertilization EC 39	VRA	70	2021	kg ha^{-1}	49	49	18	114	14.61
Fertilization EC 39	UA	34	2021	kg ha^{-1}	35				
Total fertilizer[a]	VRA	70	2021	kg ha^{-1}	147	141	104	234	17.81
Total fertilizer[a]	UA	34	2021	kg ha^{-1}	155				

[a] incl. uniform N fertilization vegetation start (50 kg ha^{-1})

Table S10. Descriptive statistics of yield and N balance parameters, field A1, winter wheat, 2021 (UA)

Parameter	n	Year	Unit	Mean	Median	Min	Max	SD
Yield	34	2021	t ha^{-1}	9.8	10.1	8.0	10.8	0.74
Grain N content	34	2021	%	1.9	1.9	1.5	2.2	0.17
Plant N uptake[a]	34	2021	kg ha^{-1}	200	198	150	245	25.34
Protein content	34	2021	%	10.8	10.6	8.6	12.7	0.95
N balance	34	2021	kg ha^{-1}	-45	-43	-90	5	25.34
N efficiency	34	2021	%	130	130	100	160	16.50

Table S11. Descriptive statistics of yield and N balance parameters, field A1, winter wheat, 2022 (VRA)

Parameter	n	Year	Unit	Mean	Median	Min	Max	SD
Yield	70	2021	t ha^{-1}	9.8	9.8	7.7	11.0	0.65
Grain N content	70	2021	%	2.0	1.9	1.6	2.3	0.14
Plant N uptake[a]	70	2021	kg ha^{-1}	204	201	152	250	21.90
Protein content	70	2021	%	11.1	11.0	9.1	13.1	0.82
N balance	70	2021	kg ha^{-1}	-61	-62	-128	3	24.84
N efficiency	70	2021	%	140	140	100	210	21.20

[a] incl. straw harvest

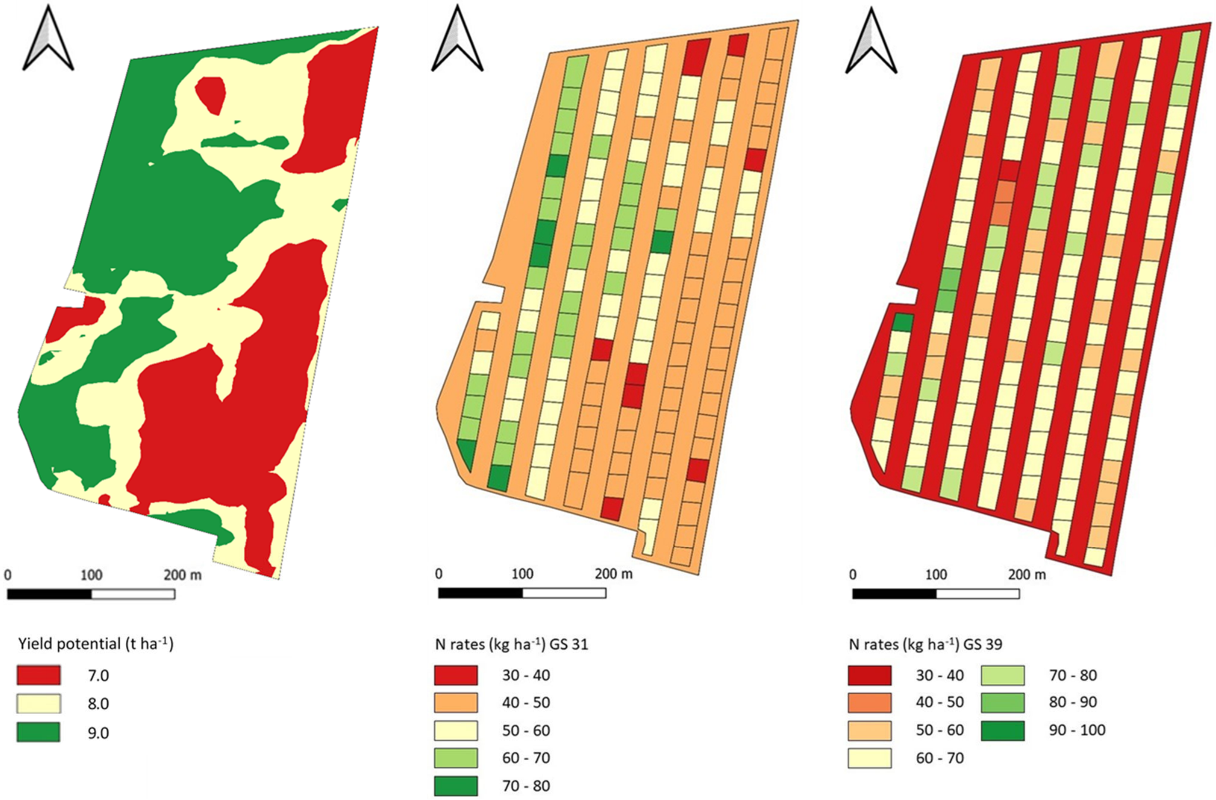

(**a**) (**b**) (**c**)

Figure S6. Yield potential map (a) and N fertilizer application maps, determined N fertilizer requirements, and applied N fertilizer application rates at growth stages GS 31 (b) and39 (c), field B1 (27 ha), winter wheat, 2022.

Table S12. Descriptive statistics of N fertilization, field B1, winter wheat, 2022

Parameter	Fertilization system	n	Year	Unit	Mean	Median	Min	Max	SD
Fertilization EC 31	VRA	100	2022	kg ha^{-1}	53	54	31	73	10.3
Fertilization EC 31	UA	122	2022	kg ha^{-1}	45				
Fertilization EC 39	VRA	100	2022	kg ha^{-1}	65	65	37	98	7.2
Fertilization EC 39	UA	122	2022	kg ha^{-1}	40				
Total Fertilizer[a]	VRA	100	2022	kg ha^{-1}	189	189	164	224	12.9
Total Fertilizer[a]	UA	122	2022	kg ha^{-1}	155				

[a] incl. uniform N fertilization vegetation start (70 kg ha^{-1})

Table S13. Descriptive statistics of yield and N balance parameters, field B1, winter wheat, 2022 (UA)

Parameter	n	Year	Unit	Mean	Median	Min	Max	SD
Yield	100	2022	t ha^{-1}	8.4	8.5	3.9	9.5	0.81
Grain N content	100	2022	%	1.7	1.6	1.4	2.6	0.24
Plant N uptake[a]	100	2022	kg ha^{-1}	154	152	107	214	18.03
Protein content	100	2022	%	10.4	9.9	8.5	16.4	1.47
N balance	100	2022	kg ha^{-1}	0.6	2.4	-59	48	18.03
N efficiency	100	2022	%	100	100	70	140	12.30

Table S14. Descriptive statistics of yield and N balance parameters, field B1, winter wheat, 2022 (VRA)

Parameter	n	Year	Unit	Mean	Median	Min	Max	SD
Yield	122	2022	t ha^{-1}	8.4	8.5	2.5	11.0	1.17
Grain N content	122	2022	%	1.7	1.6	1.5	2.7	0.22
Plant N uptake[a]	122	2022	kg ha^{-1}	159	155	70	209	22.50
Protein content	122	2022	%	10.6	10.2	9.2	17.1	1.35
N balance	122	2022	kg ha^{-1}	29	28	-25	150	26.23
N efficiency	122	2022	%	80	80	30	110	13.00

[a] incl. straw harvest

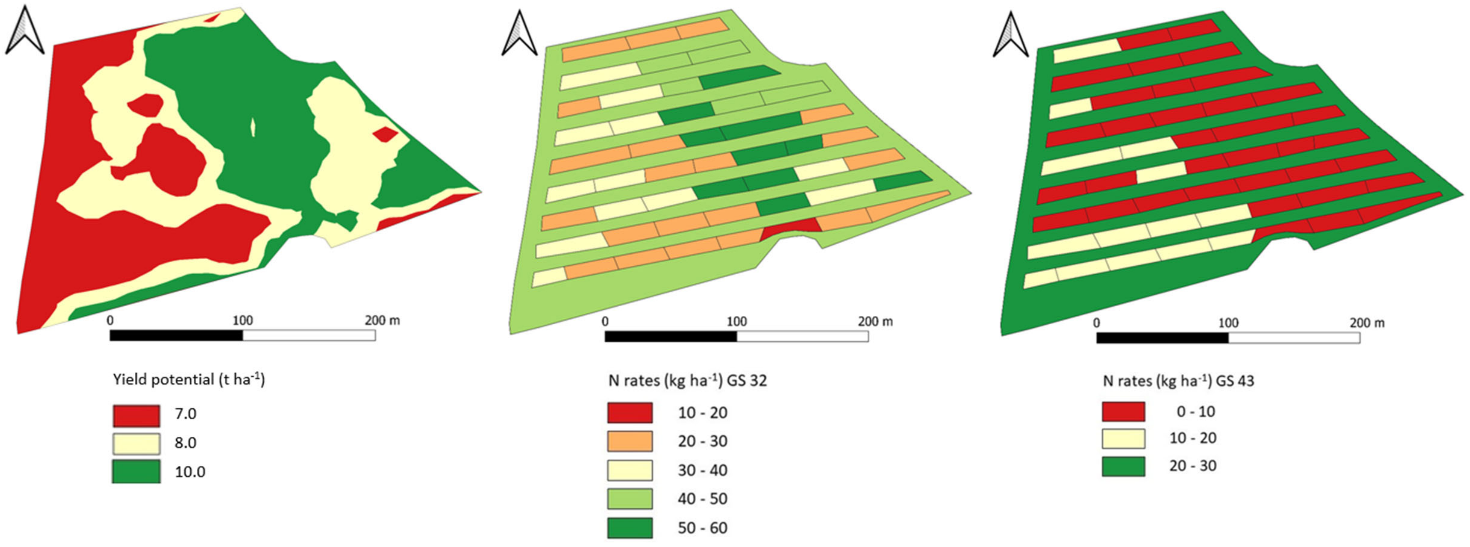

(**a**) (**b**) (**c**)

Figure S7. Yield potential map (a) and N fertilizer application maps, determined N fertilizer requirements, and applied N fertilizer application rates at growth stages 32 (b) and 43 (c), field C1 (7.5 ha), winter wheat, 2022.

Table S15. Descriptive statistics of N fertilization, field C1, winter wheat, 2022

Parameter	Fertilization system	n	Year	Unit	Mean	Median	Min	Max	SD
Fertilization EC 32	VRA	32	2022	kg ha^{-1}	37	33	17	58	11.37
Fertilization EC 32	UA	33	2021	kg ha^{-1}	50				
Fertilization EC 43[a]	VRA	32	2022	kg ha^{-1}	1	1	-17	19	9.65
Fertilization EC 43[b]	VRA	32	2022	kg ha^{-1}	5	1	0	19	5.96
Fertilization EC 43	UA	33	2021	kg ha^{-1}	30				
Total Fertilizer[c]	VRA	32	2022	kg ha^{-1}	168	166	143	206	9.90
Total Fertilizer[c]	UA	33	2021	kg ha^{-1}	206				

[a] original fertilization

[b] corrected fertilization

[c] incl. uniform N fertilization at vegetation start (126 kg ha^{-1})

Table S16. Descriptive statistics of yield and N balance parameters, field C1, winter wheat, 2022 (UA)

Parameter	n	Year	Unit	Mean	Median	Min	Max	SD
Yield	33	2022	t ha^{-1}	9.0	9.2	4.5	10.0	1.03
Grain N content	33	2022	%	2.0	2.0	1.5	2.4	0.16
Plant N uptake[a]	33	2022	kg ha^{-1}	177	182	89	207	23.19
Protein content	33	2022	%	11.3	11.3	8.5	13.6	0.93
N balance	33	2022	kg ha^{-1}	29	24	-1	117	23.19
N efficiency	33	2022	%	90	90	40	100	12.2

Table S17. Descriptive statistics of yield and N balance parameters, field C1, winter wheat, 2022 (VRA)

Parameter	n	Year	Unit	Mean	Median	Min	Max	SD
Yield	32	2022	t ha^{-1}	8.8	9.0	5.0	9.5	0.93
Grain N content	32	2022	%	1.9	1.9	1.8	2.2	0.10
Plant N uptake[a]	32	2022	kg ha^{-1}	170	172	95	194	20.81
Protein content	32	2022	%	11.1	11.0	10.3	12.5	0.60
N balance	32	2022	kg ha^{-1}	-1	-7	-31	67	21.12
N efficiency	32	2022	%	100	100	60	120	13.0

[a] incl. straw harvest

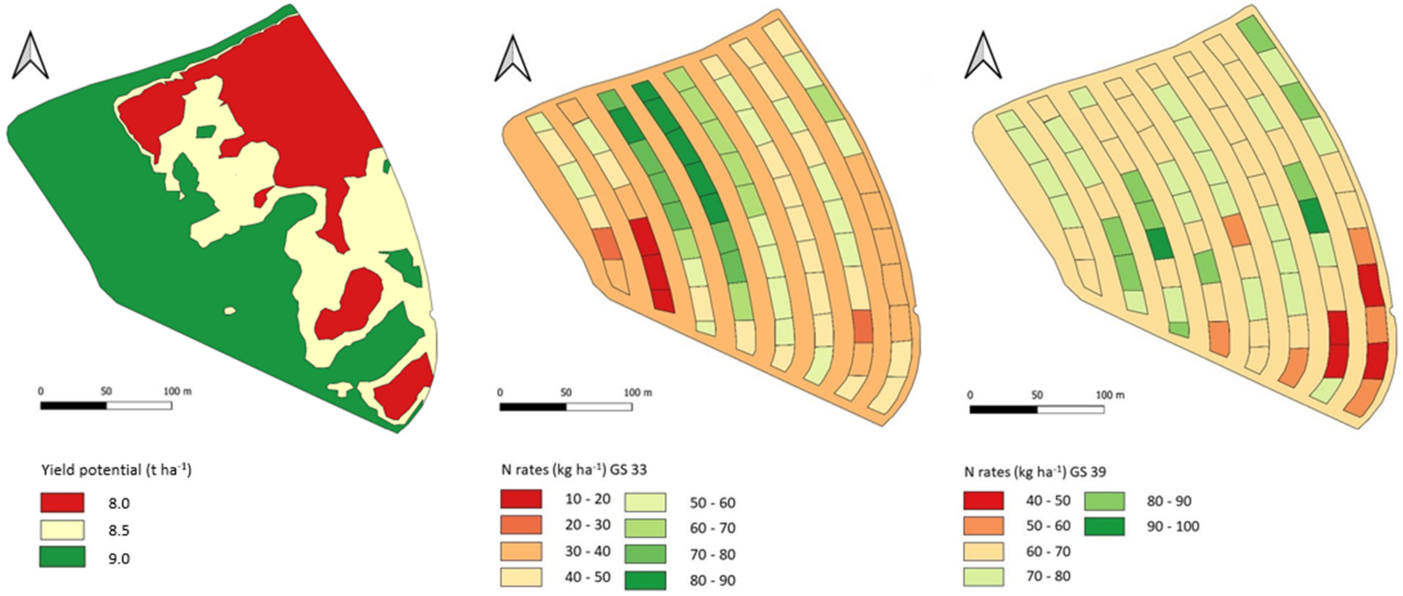

(a) **(b)** **(c)**

Figure S8. Yield potential map (a) and N fertilizer application maps, determined N fertilizer requirements, and applied N fertilizer application rates at growth stages 33 (b) and 39 (c), field C2 (9.5 ha), winter wheat, 2023.

Table S18. Descriptive statistics of N fertilization, field C2, winter wheat, 2023

Parameter	Fertilization system	n	Year	Unit	Mean	Median	Min	Max	SD
Fertilization EC 32[a]	VRA	32	2023	kg ha^{-1}	71	70	33	108	16.5
Fertilization EC 32[b]	VRA	32	2023	kg ha^{-1}	52	50	14	88	16.01
Fertilization EC 32	UA	35	2021	kg ha^{-1}	35				
Fertilization EC 39[a]	VRA	32	2023	kg ha^{-1}	77	77	46	100	11.40
Fertilization EC 39[b]	VRA	32	2023	kg ha^{-1}	70	69	42	90	10.23
Fertilization EC 39	UA	35	2021	kg ha^{-1}	70				
Total Fertilizer[c]	VRA	32	2023	kg ha^{-1}	234	237	170	274	22.36
Total Fertilizer[c]	UA	35	2021	kg ha^{-1}	217				

[a] original fertilization

[b] corrected fertilization

[c] incl. uniform N fertilization at vegetation start (112 kg ha^{-1})

Table S19. Descriptive statistics of yield and N balance parameters, field C2, winter wheat, 2022 (UA)

Parameter	n	Year	Unit	Mean	Median	Min	Max	SD
Yield	35	2022	t ha^{-1}	10.0	10.1	7.9	11.5	0.77
Grain N content	35	2022	%	1.7	1.6	1.6	1.8	0.06
Plant N uptake[a]	35	2022	kg ha^{-1}	171	170	138	197	12.49
Protein content	35	2022	%	9.4	9.3	8.8	10.4	0.38
N balance	35	2022	kg ha^{-1}	45	46	19	78	12.49
N efficiency	35	2022	%	80	80	60	90	6.30

Table S20. Descriptive statistics of yield and N balance parameters, field C2, winter wheat, 2022 (VRA)

Parameter	n	Year	Unit	Mean	Median	Min	Max	SD
Yield	32	2022	t ha^{-1}	10.0	10.1	7.9	11.6	0.94
Grain N content	32	2022	%	1.7	1.7	1.4	1.9	0.11
Plant N uptake[a]	32	2022	kg ha^{-1}	173	177	119	206	19.74
Protein content	32	2022	%	9.4	9.4	8.1	10.6	0.57
N balance	32	2022	kg ha^{-1}	68	67	10	106	22.80
N efficiency	32	2022	%	70	70	50	100	10.10

[a] incl. straw harvest

References

1. Wintersteiger. Wintersteiger Delta Plot Combine. Available online: file:///C:/Users/LudwigHagn/Favorites/Downloads/Delta_EN.pdf (accessed on 18 August 2023).
2. VDLUFA-Methodenbuch III. *Verband deutscher landwirtschaftlicher Untersuchungs- und Forschungsanstalten (VDLUFA)*: *Methode 4.1.2 Bestimmung von Rohprotein mittels DUMAS-Verbrennungsmethode. In: Handbuch der Landwirtschaftlichen Versuchs- und Untersuchungsmethodik*, 3. Auflage; VDLUFA-Verl.: Darmstadt, 2004.
3. Hagn, L.; Schuster, J.; Mittermayer, M.; Hülsbergen, K.-J. A new method for satellite-based remote sensing analysis of plant-specific biomass yield patterns for precision farming applications. *Precision Agric* **2024**.
4. tec5. Optical Spectroscopy - modern process analytical technologies. Available online: https://tec5.com/en/technology/optical-spectroscopy/ (accessed on 18 August 2023).
